# Effects of Water–Ethanol Extracts from Four *Sphagnum* Species on Gene Expression of Selected Enzymes in Normal Human Dermal Fibroblasts and Their Antioxidant Properties

**DOI:** 10.3390/ph16081076

**Published:** 2023-07-28

**Authors:** Maria Zych, Katarzyna Urbisz, Magdalena Kimsa-Dudek, Maria Kamionka, Sławomir Dudek, Barbara Klaudia Raczak, Stanisław Wacławek, Damian Chmura, Ilona Kaczmarczyk-Żebrowska, Adam Stebel

**Affiliations:** 1 Department of Pharmacognosy and Phytochemistry, Faculty of Pharmaceutical Sciences in Sosnowiec, Medical University of Silesia in Katowice, Jagiellońska 4, 41-200 Sosnowiec, Poland; kasiaurbisz@poczta.fm (K.U.); sdudek@sum.edu.pl (S.D.); izebrowska@sum.edu.pl (I.K.-Ż.); 2 Department of Nutrigenomics and Bromatology, Faculty of Pharmaceutical Sciences in Sosnowiec, Medical University of Silesia, Katowice, Jedności 8, 41-200 Sosnowiec, Poland; mkimsa@sum.edu.pl; 3 Department of Pharmaceutical Botany, Faculty of Pharmaceutical Sciences in Sosnowiec, Medical University of Silesia in Katowice, Ostrogórska 30, 41-200 Sosnowiec, Poland; ma.kamionka@gmail.com (M.K.); astebel@sum.edu.pl (A.S.); 4 Institute for Nanomaterials, Advanced Technologies and Innovation (CXI), Technical University of Liberec (TUL), Studentská 1402/2, 46117 Liberec, Czech Republic; barbara.klaudia.raczak@tul.cz (B.K.R.);; 5 Faculty of Mechatronics, Informatics and Interdisciplinary Studies, Technical University of Liberec (TUL), 46117 Liberec, Czech Republic; 6 Institute of Environmental Protection and Engineering, University of Bielsko-Biala, Willowa 2, 43-309 Bielsko-Biala, Poland; dchmura@ath.bielsko.pl

**Keywords:** antioxidant properties, collagenase, elastase, genes expression, hyaluronic acid, NHDF cell line, *Sphagnum* mosses, tyrosinase

## Abstract

Mosses (Bryophyta), particularly species of the genus *Sphagnum*, which have been used for centuries for the treatment of skin diseases and damage, are still not explored enough in terms of their use in cosmetics. The purpose of this study was to determine the antioxidant properties of water–ethanol extracts from four selected species of the genus *Sphagnum* (*S. girgenshonii* Russow, *S. magellanicum* Brid., *S. palustre* L., and *S. squarrosum* Crome) and their impact on the expression of genes encoding key enzymes for the functioning of the skin. In this study, the effects of *Sphagnum* extracts on the expression of genes encoding tyrosinase, collagenase, elastase, hyaluronidase and hyaluronic acid synthase in human dermal fibroblasts were determined for the first time in vitro. The extracts inhibited tyrosinase gene expression and showed antioxidant activity. The experiment showed an increase in the expression of some genes encoding collagenase (MMP1) or hyaluronidase (HYAL2, HYAL3 and HYAL4) and a decrease in the hyaluronan synthase (HAS1, HAS2 and HAS3) genes expression by the tested extracts. The obtained results suggest that using extracts from the tested *Sphagnum* species in anti-aging cosmetics does not seem beneficial. Further studies are needed to clarify their impact on the skin.

## 1. Introduction

The genus *Sphagnum*, belonging to the group of mosses Bryophyta, is a very distinct group of plants. They occur mainly in wetlands and water areas, e.g., peat bogs, oxbow lakes, drainage ditches or near lobelia lakes. Having the ability to store water, they maintain high humidity and appropriate acidity of the ground in the habitats where they occur, which contributes to the slow process of their decomposition and peat accumulation [1]. The number of *Sphagnum* species worldwide is estimated at approximately 350–500 [2]. There are about 58 species in Europe [3], of which 37 have been recorded in Poland [4].

Species belonging to the genus *Sphagnum* are the source of many bioactive chemical compounds. Their content in the plant depends on the season and from where the research material is collected, as well as the plant species [5]. They contain polysaccharides (e.g., a pectin-like carbohydrate polymer called sphagnan) [6,7], amino acids, carotenoids, fatty acids, triterpenes and sterols, as well as phenolic compounds including phenolic acids and flavonoids [5,8,9,10,11]. Among the phenolic acids occurring in species of the genus *Sphagnum* the presence of, among others, dihydroxybenzoic, gallic, vanillic, salicylic, caffeic, chlorogenic, *p*-coumaric and cinnamic acids [8,11], as well as sphagnic acid (characteristic of these plants) [5,8], was detected. Several publications also report the presence of a flavonoid fraction, unique for this type, containing both aglycones and flavonoid glycosides belonging, among others, to flavonols or flavanones [9,12].

The presence of many primary and secondary metabolites in plants of the *Sphagnum* genus has prompted in vitro studies investigating the extracts obtained from these plants. Scientific research confirms their antibacterial [8,11,13,14], antioxidant [11,15,16,17], cytotoxic [8,10], aromatase inhibiting activity [12] and protective effects on kidney epithelial cells [9].

The use of various species of *Sphagnum* in ethnomedicine has certainly contributed to the research mentioned above and expanded knowledge about the use of this group of plants. In traditional Chinese medicine, they are used in the treatment of skin diseases, acne, eye diseases and hemorrhoids [18], while traditional Korean medicine finds them effective in treating heart pain and strokes [9,12]. Various facts regarding the historical usage of the genus *Sphagnum* in medicine and ethnomedicine were collected and described by Drobnik and Stebel in their work [19]. The most commonly practiced use of species of the genus *Sphagnum* is observed in the treatment of skin diseases and injuries. Antimicrobial and absorption properties allowed the effective use of these plants as early as in the Mayan Civilization [20]. Since 1880, they have also been used in Europe as wound dressings and in the prevention of gas gangrene, in particular during World War I [7,18,19,21,22]. Recent studies on species of the genus *Sphagnum* revealed the UV-absorbing effect of extracts obtained from various species, such as *S. magellanicum* Brid. and *S. meridense* (Hampe) Müll.Hal., which suggests the possibility of using them or individual compounds isolated from them as natural sunscreens [15,16]. These reports suggest the need for further research to determine the possibility of using extracts from various species of *Sphagnum* as ingredients in cosmetics, especially in cosmetics intended as anti-aging treatments.

The ingredients of cosmetics (including anti-aging ones) should be characterized by antioxidant activity and should also prevent the degradation of skin components, e.g., collagen, elastin or hyaluronic acid, as well as protect against increased melanin synthesis [23,24]. Sources of ingredients with collagenase, elastase, hyaluronidase or tyrosinase inhibitory activity are constantly being sought. The results of the research on plant extracts obtained from, e.g., *Camellia sinensis* (L.) Kuntze, *Hypericum scruglii* Bacch., Brullo & Salmeri, *Limonium morisianum* Arrigoni, *Cistus salviifolius* L., *Pistacia lentiscus* L., *Cytinus hypocistis* (L.) L., *Himantoglossum robertianum* (Loisel.) P. Delforge, *Plectranthus* spp., *Salicornia europaea* L., and *Rosa rugosa* Thunb., as well as bryophytes such as *Polytrichum formosum* Hedw., indicate that they might be a source of potential inhibitors of enzymes responsible for skin aging processes [25,26,27,28,29,30].

Based on the above-mentioned literature, it can be hypothesized that species from the genus *Sphagnum* can be used as ingredients in anti-aging cosmetics. Therefore, this study aimed to evaluate the antioxidant properties of water–ethanol extracts prepared from four moss species (*Sphagnum girgensohnii* Russow, *Sphagnum magellanicum* Brid., *Sphagnum palustre* L. and *Sphagnum squarrosum* Crome) and to determine their effects on the expression of genes encoding enzymes involved in the metabolism of hyaluronic acid, collagen, elastin and melanin in human dermal fibroblasts.

## 2. Results

### 2.1. Results of Determination of the Content of Phenolic Compounds

**Figure 1:**
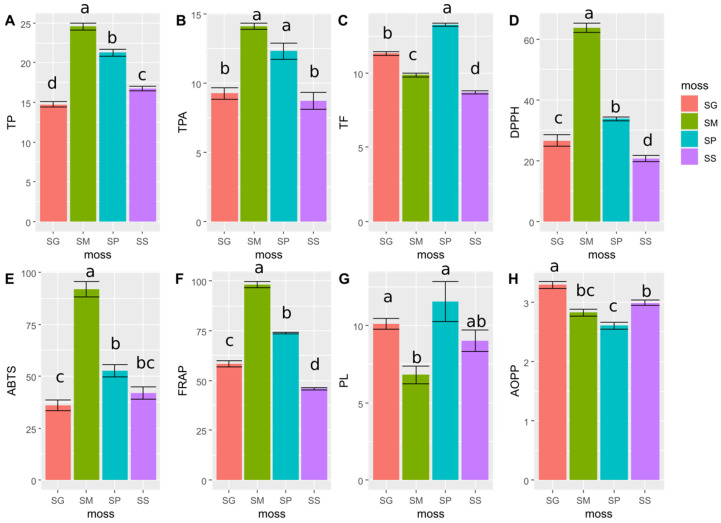
Comparison of selected variables among four moss species (SG, SM, SP, SS) based on LMM tests. The different lowercase letters (a, b, c and d) above bars denote significant differences at *p* < 0.05 (LSD Fisher test) among the control and mosses. Results are mean ± SE, n = 6. (**A**) total phenols content (TP) as mg GAE/g dry extract; (**B**) total phenolic acids content (TPA) as mg CAE/g dry extract; (**C**) total flavonoids content (TF) as mg QE/g dry extract; (**D**) antioxidants activity measured by DPPH method as µM TE/g dry extract; (**E**) antioxidants activity measured by ABTS method as µM TE/g dry extract; (**F**) antioxidants activity measured by FRAP method as µM Fe^2+^E/g dry extract; (**G**) lipid peroxidation (PL) as % inhibition of linoleic acid oxidation; (**H**) advanced oxidation protein products (AOPP) as µmol ChTE/mL.

The results of the determination of total phenols content (TP), total phenolic acids content (TPA) and total flavonoids (TF) are shown in Figure 1A–C, respectively (Figure 1). The highest TP was 24.6 ± 1.1 mg GAE/g dry extract, which was observed in the SM extract. TP decreased in the extracts in the order of SM > SP (21.28 ± 1.07 mg GAE/g dry extract) > SS (16.73 ± 0.73 mg GAE/g dry extract) > SG (14.75 ± 0.87 mg GAE/g dry extract). The extracts from the SM and SP species were distinguished by the highest TPA, which were 14.14 ± 0.56 and 12.33 ± 1.42 mg CAE/g dry extract, respectively. Significantly lower TPA were recorded for SS (8.75 ± 1.49 mg CAE/g dry extract) and SG (9.28 ± 1.01 mg CAE/g dry extract) species. In turn, the highest TF was 13.27 ± 0.24 mg QE/g dry extract, which was observed for the SP extract. The amount of TF decreased in the order SP > SG (11.33 ± 0.31 mg QE/g dry extract) > SM (9.88 ± 0.31 mg QE/g dry extract) > SS (8.70 ± 0.27 mg QE/g dry extract).

Using the HPLC technique, selected phenolic compounds were determined in extracts from the tested mosses. In all examined *Sphagnum* extracts, *p*-coumaric acid and rutin were observed. Quercetin was also detected in the *Sphagnum magellanicum* (SM) extract, and apigenin was detected in the *Sphagnum squarrosum* (SS) extract. The values of phenolic compound content are presented in Table 1. (Table 1; Figures S1–S4 in Supplementary Materials).

### 2.2. Results of the Antioxidant Properties of Moss Extracts

**Figure 2:**
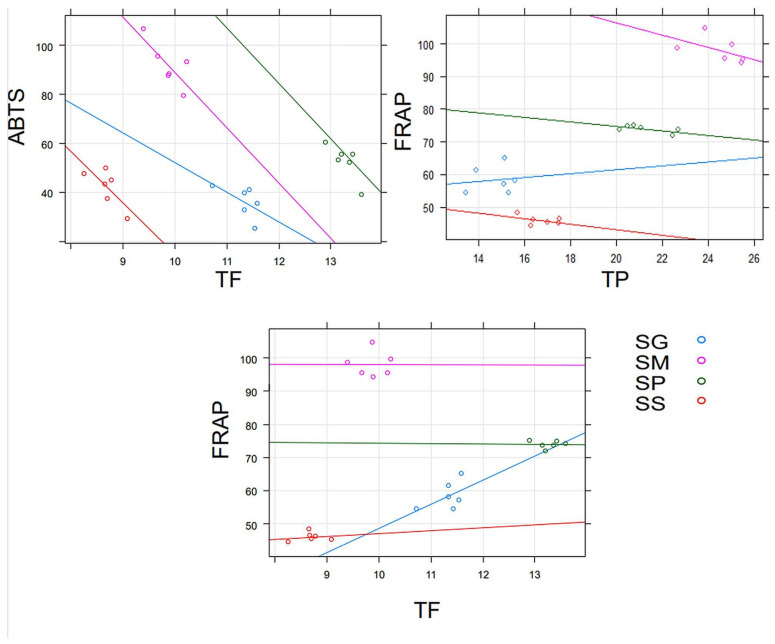
The relationships between TF, TPA, TP and antioxidant properties determined by the ABTS and FRAP methods. Only significant results are shown. For details see Supplementary Materials Table S1.

According to the LMMs, the species identity of *Sphagnum* had an impact on antioxidant properties, as determined by the ABTS, DPPH, FRAP methods, and on PL and AOPP creation (Figure 1, Supplementary Materials Table S1). The studied species also differed in TP, TPA and TF. The determined values of TP, TPA and TF also had an influence on selected antioxidant properties (Figure 2, Supplementary Materials Table S1).

The strongest antioxidant activity in the studies based on the ABTS, DPPH and FRAP methods was found for the SM extract. The antioxidant activity values measured by these methods for the SM extract were 91.78 ± 9.17 µM TE/g dry extract, 63.79 ± 3.84 µM TE/g dry extract and 98.00 ± 3.89 µM Fe^2+^E/dry extract, respectively. The lowest antioxidant activity was 20.64 ± 2.49 µM TE/g dry extract and 45.94 ± 1.38 µM Fe^2+^E/dry extract for the SS extract, based on determinations by DPPH and FRAP methods, respectively. The antioxidant potential of the SS extract measured by the ABTS method was 41.94 ± 7.60 µM TE/g dry extract, and it was not statistically different from the results obtained for SP (52.50 ± 7.21 µM TE/g dry extract) or SG (36.06 ± 6.56 µM TE/g dry extract). The results of antioxidant activity of all the tested extracts obtained from the analyzed species of *Sphagnum* are presented in Figure 1D–F (Figure 1).

The strongest inhibitory effect on the oxidation of linoleic acid (inhibiting lipid peroxidation) was shown by the SP and SG extracts, and the inhibition percentage for these species was 11.53 ± 3.16 and 10.09 ± 0.82, respectively. The weakest inhibitory effect on the oxidation of linoleic acid was found for the SM extract, with a inhibition percentage of 6.80 ± 1.40. The extract from SS had 9.02 ± 1.67 inhibition percentage, which was not statistically different from the results obtained for other tested species (Figure 1).

The strongest ability of protein oxidation inhibition, which is indicated by the reduced production of AOPP, were found for the extract obtained from SP (2.61 ± 0.14 µmol ChTE/mL). The SG extract showed the weakest ability for inhibiting AOPP formation (3.29 ± 0.13 µmol ChTE/mL (Figure 1).

There was a negative relationship between the TF content and antioxidant properties, as determined by the ABTS method, and the highest effect was caused by *S. magellanicum* and *S. palustre*. The negative relationship was found also between antioxidant capacity measured by FRAP method and TP; however, there was a slight positive effect of *S. girgensohnii*. A marginally significant positive relationship was observed between FRAP and TF (Supplementary Materials Table S1) and was chiefly found in *S. girgensohnii* extract (Figure 2).

### 2.3. Results of the Cytotoxicity Study of Extracts from Four Sphagnum Species

**Figure 3:**
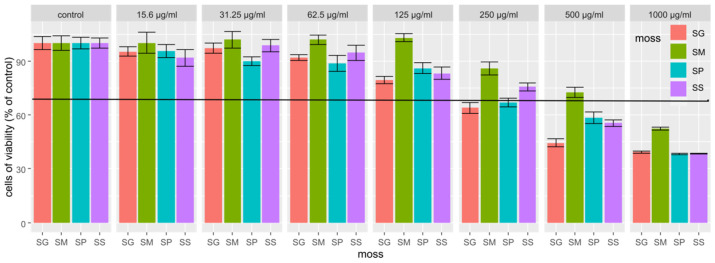
NHDF cells viability after used SG, SM, SP and SS extracts at specific concentrations. Results are mean ± SE, n = 8.

The cytotoxicity results obtained for the extracts showed that the extract from *Sphagnum magellanicum* had the lowest cytotoxicity, since even at the concentration of 500 µg/mL of the extract, the cell viability was higher than 70%. No cytotoxic effect was found for any tested extracts at the concentration of 125 µg/mL; therefore, this concentration was selected to study the gene expression of selected skin enzymes (Figure 3).

### 2.4. Results of Genes Expression Determination

**Figure 4:**
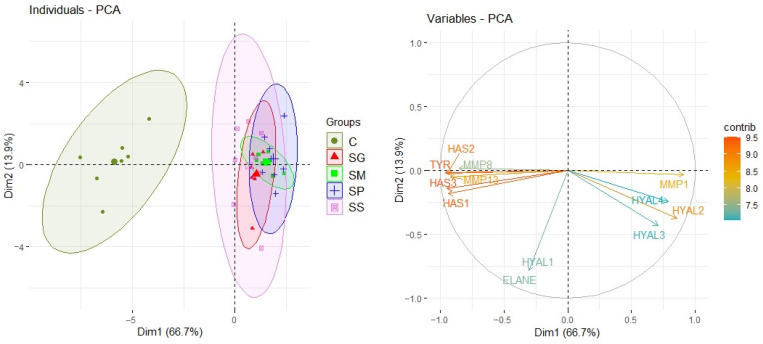
The differences among groups (control and mosses) based on relative mRNA gene expression.

**Figure 5:**
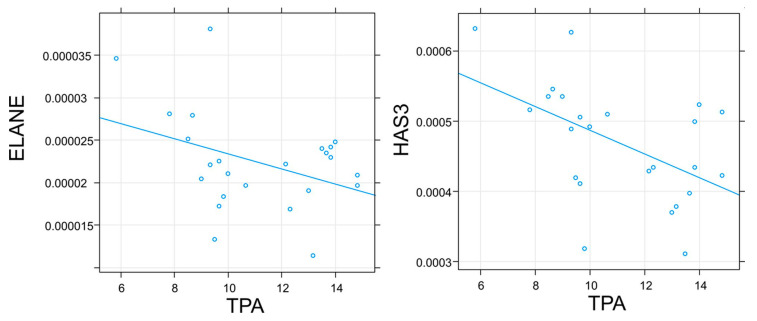
The relationship between TPA and relative mRNA expression of genes encoding human enzymes. Only significant results are shown. For details, see Supplementary Materials Table S2.

The control distinctly differed from the studied moss species in terms of relative mRNA gene expression (Figure 4). The TPA had a negative effect on the ELANE and HAS3 relative mRNA genes expression (Figure 5).

Considering moss species identity, in 10 out of 12 studied genes, there were significant differences in expression between the studied taxa and control. The extracts from all tested species of *Sphagnum* significantly increased the expression of the HYAL2, HYAL3 and HYAL4 genes compared with NHDF not treated with extracts.

The effect of the tested extracts from the genus *Sphagnum* on the relative expression of the HYAL3 gene was not statistically different between tested mosses. For the HYAL 2 and HYAL 4 genes, the lowest expression was observed after treating the NHDF with the SS extract, and this expression was still significantly higher than the expression of these genes in cells which were not treated by extracts. No impact of tested extracts on HYAL 1 expression was observed.

The tested extracts had various impacts on the expression of genes encoding collagenases (MMPs), i.e., they increased the expression of MMP1 and inhibited MMP8 and MMP13 compared with cells not treated with the extracts. No statistically significant differences between the *Sphagnum* species in the effect on the expression of these genes were observed.

The relative expression of tyrosinase gene expression (TYR) was statistically significant. Its expression was inhibited by all of the tested extracts from *Sphagnum* species in comparison to the relative expression of this gene in cells which were not treated by the extracts. The strongest, statistically significant inhibition of TYR gene expression was observed for the SP extract.

**Figure 6:**
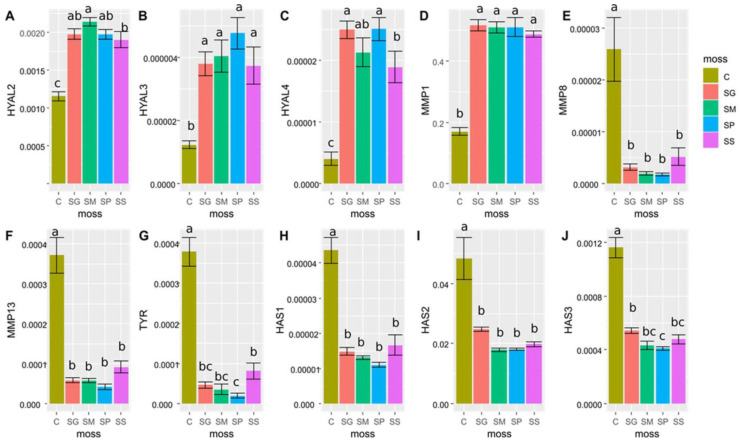
The comparison of relative mRNA expression of genes among control C and mosses (SG, SM, SP, SS). The different lowercase letters (a, b and c) above bars denote significant differences at *p* < 0.05 (LSD Fisher test) among control and mosses. Results are mean ± SE, n = 7–8. Relative mRNA expression of (**A**) HYAL2; (**B**) HYAL3; (**C**) HYAL4; (**D**) MMP1; (**E**) MMP8; (**F**) MMP13; (**G**) TYR; (**H**) HAS1; (**I**) HAS2; and (**J**) HAS3.

Extracts from all tested mosses inhibited the relative expression of hyaluronan synthases genes (HAS1, HAS2 and HAS3) in comparison with their expression in NHDF not treated by the extracts. No statistically significant differences between the *Sphagnum* species in the effect on the expression of HAS1 and HAS2 genes were observed; however, expression of the HAS3 gene was most inhibited by the SP extract (Figure 6).

## 3. Discussion

Mosses have been used in ethnomedicine for centuries, and in several extensive review papers, a wide range of their applications is presented [31,32,33]. They are used, among other applications, in skin disease, infection or cancer treatment [31,32,33]. In recent years, studies to confirm these uses of mosses and to discover new applications have been conducted. Most often, in vitro tests were conducted to determine the activity of moss extracts or active compounds isolated from them. According to our knowledge, there are no in vivo studies or clinical studies describing the activity of mosses extracts. In the literature, we found only one clinical trial description in which a galactosidase inhibitor isolated from biotechnologically recombined moss—*Physcomitrella patens*—was used [34]. An aqueous cell extract biotechnologically produced from the same moss is also a cosmetic ingredient [35]. To the best of our knowledge, there is no reported research describing the adverse effects of moss extracts. Only allergic reactions have been reported, but these reports were referring to liverworts, which are plants related to mosses [32,33]. Therefore, it is necessary to conduct research on the action of mosses extracts, and to publish results indicating both their beneficial and unfavorable activities.

The skin is the body’s largest organ, protecting against harmful chemical factors, the influence of UV radiation, penetration of microorganisms and mechanical damage to internal organs. It consists of the epithelium, dermis and subcutaneous tissue [36,37]. The dermis contains the most essential components for the skin and its functions, such as fibers of collagen and elastin, which are produced by fibroblasts, and glycosaminoglycans located between the fibers, such as hyaluronic acid. These components are the main ingredients of the ECM [38,39]. The impact of UV radiation, chemicals and pollution on the skin causes the overproduction of reactive oxygen species within it, which in turn, increases the level of oxidative stress. Increased production of free radicals leads to accelerated skin aging associated with a decline in the amounts of collagen and elastin fibers, reduction in skin thickness, and formation of wrinkles, skin cancers or hyperpigmentation (melasma, freckles, age spots) [25,27,40].

Therefore, compounds with antioxidant activity, particularly phenolic compounds, are used in cosmetics to reduce oxidative damage [41]. Due to the fact that most phenolic substances are extracted using a mixture of alcohol and water [42,43], a mixture of ethanol and water in a ratio of 1:1 was used to prepare extracts from four species of *Sphagnum*. The highest TP and TPA were found in the *Sphagnum magellanicum* extract, while the *Sphagnum palustre* extract had the highest TF. *Sphagnum magellanicum* extract also showed the strongest neutralizing effect on DPPH and ABTS radicals, as well as antioxidant properties measured by the FRAP method, while *Sphagnum palustre* extract most strongly inhibited lipid oxidation and protein oxidation. The antioxidant properties of *Sphagnum* extracts determined by the DPPH, ABTS or FRAP methods have already been described [11,15,16,17]. It is worth noting that this study is the first to compare the inhibition of biomolecules oxidation by extracts from different species of the genus *Sphagnum*. The inhibitory effect on the formation of AOPP has already been demonstrated for some phenolic acids and flavonoids including quercetin [44], which was determined in an extract of *Sphagnum magellanicum*. It is worth noting that rutin, which is a quercetin glycoside (rutinoside), was detected in all tested extracts. Some studies show that the greater the total content of phenolic substances in the extract, the greater its ability to inhibit protein oxidation and lipid peroxidation [45]. Positive correlations are often shown between the content of phenolic components and the neutralization of ABTS and DPPH radicals or antioxidant properties determined by the FRAP method [46,47]. In this experiment, no significant correlations were found between the content of phenolic compounds in water–ethanol extracts from *Sphagnum* and their ability to inhibit the formation of AOPP and oxidation of linoleic acid or DPPH radical scavenging. Klavina et al. also found no correlation between antioxidant properties and the content of phenolic compounds in several species of mosses and suggested that the antioxidant activity in mosses may result from the presence of other groups of active compounds present in Bryophyta [43]. Surprisingly, this experiment observed a negative correlation between the TF and the antioxidant activity measured by the ABTS method. A negative relationship was also noted between the iron ion reducing capacity (FRAP) and TP for the SM, SP, and SS extracts. Only the SG extract showed a positive correlation. A positive relationship was also found between the antioxidant properties of extracts determined by the FRAP method and the TF. According to some authors, the presence or absence of a relationship between the content of phenolic substances and antioxidant properties may depend on the extraction conditions [48,49].

This study demonstrates the antioxidant effect of water–ethanol extracts from four species of *Sphagnum* with a high content of polyphenols. Based on existing reports suggesting that polyphenols may be modulators of enzymes responsible for the skin’s condition, an attempt was made to determine the effects of these extracts on genes encoding enzymes such as tyrosinase, collagenase, elastase, hyaluronidases and hyaluronan synthases [23].

In order to study the effect of the obtained extracts on enzymes related to the skin condition, the cytotoxicity of these extracts was firstly tested. According to previous studies, an extract is characterized as lacking a cytotoxic effect on cells when the cells viability after its use is ≥70% (ISO 10993-5). In vitro tests are considered as a key tool for determining the cytotoxicity of extracts containing new chemical compounds. Many cell lines can be used for this purpose, among which the fibroblast line is often used [50]. Our results showed that the SM extract had the lowest cytotoxicity. It is worth noting that SM is one of the most frequently used moss species in studies [11,15,51].

Tyrosinase is a copper-dependent enzyme that is widespread in various organisms and plays a key role in melanogenesis [52]. Therefore, tyrosinase inhibitors are considered to be potentially useful components of depigmenting cosmetics [53]. Many published papers have reported the inhibition of tyrosinase activity due to plant extracts [28,29], including extracts obtained from the mosses *Hypnum cupressiforme* Hedw. [54] and *Polytrichum formosum* Hedw. [27]. Studies on the effect of plant extracts on tyrosinase activity are often based on spectrophotometric measurements, and their results indicate the inhibition of tyrosinase activity, among others by flavonoids [27,55]. However, there is evidence that flavonoids and their reactions may influence such an analysis. Tyrosinase activity is usually determined by the spectrophotometric method by measuring the absorbance of dopachrome, which is formed as a result of non-enzymatic transformations (cyclization and oxidation) of dopaquinone, the main product of reactions catalyzed by tyrosinase. Flavonoids can affect these non-enzymatic reactions and cause errors in the results [56,57].

It is also believed that mushroom tyrosinase (mostly used for screening based on spectrophotometric measurements) is not as selective as mammalian tyrosinase, and experiments conducted using mammalian cell lines seem to be a better research model than those based solely on enzymes and spectrophotometric measurements [58]. Considering these findings, human dermal fibroblasts were used in the work presented here to determine the impact of moss extracts on enzymes related to skin condition. The results of many previous studies indicate that the activity of the tyrosinase enzyme is also dependent on the presence of antioxidants, in particular phenolic compounds. In vivo studies indicate an increase in tyrosinase activity caused by the presence of free radicals (fibroblast tyrosinase). It was found that *p*-coumaric acid, thanks to its structure similar to L-tyrosine, can competitively inhibit tyrosinase [59]. In this study, *p*-coumaric acid was present in each of the tested extracts, and all the extracts were rich in antioxidant phenolic compounds. The results of the tyrosinase gene expression study indicate that the extracts obtained from all the tested *Sphagnum* species inhibit the expression of this gene compared with its expression in cells not treated with the extract. However, to strictly determine the effect of *Sphagnum* extracts on melanin synthesis, further research on other cell lines which synthesize melanin (melanocytes or melanoma cells) or in vivo studies are necessary.

Collagenases belong to the MMPs family and their activity is dependent on the zinc cation (Zn^2+^). By degrading collagen, these endopeptidases play a major role in generating structural changes in the extracellular matrix of the skin [27,37]. The following collagenases are distinguished: collagenase 1, collagenase 2, and collagenase 3. These are encoded by the genes MMP1, MMP8 and MMP13, respectively [37,39]. The most important role in collagen metabolism is played by collagenase 1 (MMP1), also known as interstitial or fibroblast collagenase [60,61,62]. Unfortunately, extracts from the studied *Sphagnum* species in this study increased the expression of MMP1 genes, which may contribute to increased degradation of collagen in the skin and intensification of skin aging processes. Although *Sphagnum* extracts reduced gene expression of MMP8 and MMP13 in cells which were treated by extracts, compared with gene expression in dermal fibroblasts to which *Sphagnum* extracts were not added, this effect seems to be less significant, since MMP8 and MMP13 are thought to contribute to a very small extent to the overall damage to the collagen structure in the photoaging process [37,39].

In addition to collagen, an important protein in the ECM of mammals is elastin. It provides flexibility and elasticity to various tissues and organs, including the skin. Elastin is broken down by enzymes such as serine proteases, MMPs and cysteine proteases [62,63]. In this study, the effects of *Sphagnum* extracts on the expression of the ELANE gene, which encodes the enzyme of neutrophil elastase [64], a serine protease [63], were analyzed. Some authors point to the important (greater than fibroblast elastase) role of neutrophil elastase in the process of elastin decomposition in the skin [65]. In this study, no effect of *Sphagnum* extracts on the neutrophil elastase (ELANE) gene expression was observed compared with the expression of the gene in fibroblasts not treated with the extracts, and there was a negative correlation between gene expression and TPA in the extracts. Elastase is, therefore, an enzyme whose activity depends on the presence of antioxidants. Another study also confirmed the relationship between the decrease in elastase activity along with the increase in the content of antioxidants, including phenolic compounds [66].

HA is an essential component of the ECM and is chemically a non-sulphated glycosaminoglycan. It has an impact on tissue homeostasis, in particular on the formation of intercellular gels and facilitating the appropriate organization of tissues. Half of the total amount of HA in the human body is found in the skin (epidermis and dermis), where its synthesis by epidermal keratinocytes and dermal fibroblasts occurs [67,68]. HA with high molecular weight is present in normal tissues, while HA with lower molecular weight is responsible for scar formation, inflammation, immunostimulation and angiogenesis. HA synthesis occurs with the participation of hyaluronic synthases (HAS), which are glycosyl transferases. Three isoforms of HAS have been identified in mammals, where fibroblasts mainly use HAS2, while keratinocytes equally use HAS2 and HAS3 [67]. Decreased HAS expression and reduced hyaluronic acid deposition have been reported in aging fibroblasts. Degradation of HA occurs with the participation of hyaluronidases (HYAL), with the most active ones being HYAL1 and HYAL2 [67]. In this study, a decrease in the expression of the HAS1, HAS2 and HAS3 genes and increases in the expression of the HYAL2, HYAL3 and HYAL4 genes in human dermal fibroblasts under the influence of water–ethanol extracts from all four tested *Sphagnum* species were observed compared with the expression of these genes in fibroblasts not treated with the extracts. Despite the lack of influence of the extracts on the expression of the main gene encoding hyaluronidase (HYAL1), reduced expression of HAS genes entails reduced synthesis of hyaluronic acid, and increased expression of genes from the HYAL family leads to its faster degradation. Therefore, the results of the present study appear to show the adverse effect of *Sphagnum* extracts on HA transformations in human fibroblasts, which may result in a reduced HA content in the skin. However, it is difficult to compare the results of the above work with other data in the literature because to date, there are no studies showing the effect of water–ethanol extracts from mosses of the genus *Sphagnum* on the content of HA in the skin by affecting the expression of genes encoding enzymes involved in its transformation.

During skin aging processes, the composition of the ECM changes. First of all, collagen, skin’s basic building protein, undergoes fragmentation in which MMPs play the greatest role [38,69]. The aging-related increase in the activity of MMPs may be due to the reduction in the size of fibroblasts in old skin compared with young skin [38,70]. In addition, in aging skin, the level of endogenous MMPs inhibitors may decrease, which may lead to increased MMPs activity and collagen breakdown [38]. In the process of natural aging, the number of elastin fibers decreases [38]. Data from the literature indicate no differences in the level of HA between young and old skin, but naturally aging skin possesses lower amounts of HA binding proteins than young skin [38,71]. Therefore, in research on the anti-aging properties of cosmetic ingredients such as plant extracts, it is worth determining their effect on the expression of ECM components. In this study, the expression levels of enzymes’ genes which are involved in the metabolism of ECM components were determined, but the expression of specifically these components, i.e., collagen, elastin and HA, was not. The lack of these determinations is one of the limitations of this study. Another is the lack of research using other cell lines, such as NHEK (Normal Human Epidermal Keratinocytes), melanocyte or melanoma cells. Our study is a preliminary study, providing a good basis for further research due to the unusual results obtained.

## 4. Materials and Methods

### 4.1. Mosses and Extracts Preparation

In southern Poland (49°32′15″ N 19°20′09″ E), in the Pilsko massif located in the Western Carpathians, on 30 May 2021, gametophytes of four *Sphagnum* species were collected: *Sphagnum girgensohnii* Russow. (SG), *Sphagnum magellanicum* Brid. (SM), *Sphagnum palustre* L. (SP), and *Sphagnum squarrosum* Crome (SS). Table 2 presents photographs and brief characteristics of these species. Voucher specimens were deposited in the Herbarium of the Department of Pharmaceutical Botany of Medical University of Silesia in Katowice.

The mosses’ gametophytes were dried. A 2 g sample of the powdered material was extracted with 50 mL of a mixture of ethanol and water (1:1 *v/v*) for half an hour in a round bottom flask under a reflux condenser at a temperature of 80 °C. The extract was then filtered through cotton wool. The precipitate together with the cotton wool was returned to the flask, 50 mL of the mixture of ethanol and water in the same volume ratio was added, and the extraction was repeated for another 30 min. After this time, the extract was filtered and combined with the extract obtained in the first stage of extraction. The extracts obtained in this way were concentrated on an evaporator under lowered pressure and then lyophilized. Lyophilized extracts were used to prepare solutions with a concentration of 1 mg/mL, which were then used for further research. To determine the phenolic compounds and antioxidant properties, dry (lyophilized) extracts were dissolved in a mixture of 96% ethanol and water (1:1 *v/v*). Dry extracts dissolved in water were used to investigate the impact of the moss extracts on AOPP formation. Two independent extracts were performed for each species of moss, and three determinations were performed on each extract. In the cell culture studies, the dry extracts were dissolved directly in the culture medium.

### 4.2. Equipment, Materials and Reagents Used in the Determination of Phenolic Compounds and Antioxidant Activity

#### 4.2.1. Equipment and Materials

The following equipment was employed: an HLP 5sp demineralizer (Hydrolab, Straszyn, Poland); a microplate reader Tecan Infinite M200 PRO with Magellan 7.2 software (Tecan Austria, Grödig, Austria); Spectra/Por dialysis membranes 10 kDa MWCO (Spectrum Laboratories, Phoenix, AZ, USA); and 96-well plates (Greiner, Frickenhausen, Germany).

#### 4.2.2. Reagents

The following reagents were bought from Sigma-Aldrich (St. Louis, MO, USA): 2,2′-azino-bis(3-ethylbenzothiazoline-6-sulfonic acid) (ABTS); 2,2-diphenyl-1-picrylhydrazyl (DPPH); 2,4,6-Tris(2-pyridyl)-s-triazine (TPTZ); acetonitrile; aluminium trichloride (AlCl_3_); apigenin; ascorbic acid; bovine serum albumin (BSA); caffeic acid; chloramine T; gallic acid; linoleic acid; PBS; potassium persulfate; quercetin; and rutin, trolox and thiobarbituric acid. The following were purchased from CHEMPUR (Piekary Śląskie, Poland): 96% ethanol (46.07 g/mol, 0.808 g/mL); Arnov’s reagent; hydrochloric acid (HCl); iron sulfate (FeSO_4_); methanol; and sodium acetate (CH_3_COONa). Reagents such as calcium carbonate (CaCO_3_), iron trichloride (FeCl_3_) and sodium carbonate (Na_2_CO_3_) were manufactured by POCH (Gliwice, Poland). Folin–Ciocalteau reagent and trichloroacetic acid were manufactured by Eurochem BGD (Tarnów, Poland). Sodium hydroxide (NaOH) was purchased from STANLAB (Lublin, Poland) and *p*-coumaric acid from Fluka (Chemie AG, Buchs, Switzerland). Orthophosphoric acid (H_3_PO_4_; 85%) was delivered by Penta (Prague, Czech Republic).

### 4.3. Cell line, Equipment, Materials and Reagents Used for Cell Culture and in Molecular Research

Normal human dermal fibroblasts (NHDF) cell line was obtained from Clonetics (CC-2511; San Diego, CA, USA).

#### 4.3.1. Equipment and Materials

The following equipment was employed: Countess TM Automated Cell Counter (Invitrogen, Carlsbad, CA, USA); Incubator CB53 (Binder, Tuttlingen, Germany); LightCycler^®^ 480 Instrument II (Roche Life Science, Basel, Switzerland); Microplate reader Wallac 1420 VICTOR (PerkinElmer, Waltham, MA, USA); Spectrophotometer MaestroNano MN-913 (MaestroGen Inc., Las Vegas, NV, USA); 25 cm^2^ cell culture flasks (Sarstedt, Nümbrecht, Germany); and 96-well culture plates (Nunc, Wiesbaden, Germany).

#### 4.3.2. Reagents

The following reagents were obtained: FGM-2 Fibroblast Growth Medium-2 Bullet Kit (Lonza, Basel, Switzerland); 3-[4,5-dimethylthiazol-2-yl]-2,5-diphenyltetrazolium bromide (MTT, Sigma-Aldrich, St Louis, MO, USA); PBS (Lonza, Basel, Switzerland); dimethyl sulfoxide (Sigma-Aldrich, St Louis, MO, USA); trypan blue (Invitrogen, Carlsbad, CA, USA); TRIzol reagent (Invitrogen, Carlsbad, CA, USA); SensiFAST SYBR No-ROX One-Step (Bioline, London, UK); and oligonucleotide specific primers (Sigma-Aldrich, St Louis, MO, USA).

### 4.4. Determination of the Phenolic Compounds Content

#### 4.4.1. TP Determination

Extract samples (1 mg/mL) of 20 µL and gallic acid standard solutions at 0–100 µg/mL concentrations were added to the appropriate wells of the 96-well plate. Then, 100 µL of 10% Folin–Ciocalteau reagent was added to each well and mixed. After 6 min of incubation at room temperature, 80 µL of 7.5% Na_2_CO_3_ was added to each well, mixed and incubated for 2 h. After this time, the absorbance at 740 nm was measured using a microplate reader. TP was expressed as mg GAE/g dry extract [54].

#### 4.4.2. TPA Determination

Extract samples (1 mg/mL) of 10 µL and caffeic acid standard solutions at concentrations of 0–100 µg/mL were added to the appropriate wells of a 96-well plate. Then, 20 µL of Arnova reagent, 20 µL of 0.1 M hydrochloric acid, 20 µL of 1 M sodium hydroxide, and 120 µL of distilled water were added to each well. After shaking the plate for 30 s, the absorbance at 490 nm was read using a microplate reader. TPA was expressed as mg CAE/g of dry extract [54].

#### 4.4.3. TF Determination

The modified method described by Mihailović et al. [72] was used to determine the total content of flavonoids. The modification was based on reducing the volume of the samples and reagents, and the measurement of absorbance was performed in a 96-well plate using a microplate reader. Extract samples (1 mg/mL) of 100 µL and quercetin standard solutions at concentrations of 0–100 µg/mL were added to the appropriate wells in a 96-well plate, and then 100 µL of a 2% methanolic solution of aluminum trichloride (AlCl_3_) was added and incubated at room temperature for 1 h. The absorbance at 415 nm was then measured using a microplate reader. TF was expressed as mg QE/g of dry extract.

#### 4.4.4. Determination of Selected Phenolic Compounds Using the HPLC Method

The content of selected phenolic acids (gallic acid, 4-coumaric acid) and flavonoids (quercetin, rutin and apigenin) was determined in the dilutions of dry extracts of the tested mosses with a final concentration of 1 mg/mL. These were prepared by dissolving the dry extracts in a mixture of 96% ethanol and water (1:1 *v/v*). An HPLC apparatus (UltiMate 3000; Thermo Fisher Scientific, Prague, Czech Republic) with a UV-VIS (VWD-3100) detector was used for the analysis. The compounds were separated on the C18 column (150 mm × 4.6 mm, 2.6 μm; Phenomenex, Prague, Czech Republic) and heated to 40 °C. The injection volume and flowrate were set to 20 µL and 1.0 mL/min, respectively. As for the mobile phase, it consisted of (A) DI water (pH of 3 adjusted by H_3_PO_4_) and (B) acetonitrile. The linear gradient program was used: 0.0–3.0 min 10% (B), 3.0–13.0 min 80% (B), 13.0–14.0 min 80% (B), and 14.1–17.0 min 10% (B). A wavelength of 272 nm was used for the detection of all compounds. The limit of detection (LOD), limit of quantitation (LOQ), standard error and linearity range were calculated for each compound in mg/L. The LOD and LOQ were established using standard methods according to Knoll [73] as 3 and 10 times the standard deviation of the baseline noise, respectively. The standard error was calculated from the standard error of the y-intercept. When calibrating the chemicals, the maximum concentration was 100 mg/L; therefore, the range was established from LOQ to 100 mg/L. All the data are presented in Table A1 (Appendix A).

### 4.5. Determination of the Antioxidant Properties of Moss Extracts

#### 4.5.1. DPPH Method

The determination of antioxidant properties by means of the DPPH method was carried out using the protocol described by Bobo-García et al. [74]. Extract samples (1 mg/mL) of 20 µL and 0–100 µM trolox standard solutions were added to the appropriate wells of a 96-well plate. Then, 180 µL of DPPH solution at a concentration of 150 µmol/L in methanol–water (80:20, *v/v*) was added to each well and shaken for 60 s. After incubation in the dark for 40 min, the absorbance at 515 nm was measured. The antioxidant properties results are expressed as µM TE/g extract.

#### 4.5.2. ABTS Method

The determination of antioxidant properties using the ABTS method was carried out on the basis of a modified method from Re et al. [75]. Firstly, a 7 mM ABTS solution and a 2.45 mM potassium persulfate solution were prepared. Then, these solutions were mixed and left in the dark for 12–16 h at room temperature to obtain the ABTS radical cation (ABTS•+). The obtained solution was diluted with 96% ethanol in order to obtain an absorbance of 0.70 ± 0.02 at 734 nm. Then, 4 μL of extract samples (1 mg/mL), trolox standard solutions at a concentration of (0–100 μM), and 200 μL of the diluted radical cation solution ABTS were placed in the appropriate wells of the 96-well plate, and the plate was shaken. The absorbance was estimated at 734 nm after 6 min of incubation. The results are shown as µM TE/g extract.

#### 4.5.3. FRAP Method

The iron-reducing antioxidant potential (FRAP) was determined based on a modified method described by Benzie and Strain [76]. The working reagent was prepared directly before use by mixing 300 mM of acetate buffer (pH = 3.6), 10 mM TPTZ in 40 mM HCl, and 20 mM of FeCl_3_·6H_2_O in a volume ratio of 10:1:1. Then, 10 μL of extract samples (1 mg/mL), standard FeSO_4_ solutions at concentrations in the range 0–100 μM, and 300 μL of working solution were added to the appropriate wells of a 96-well plate, and the plate was shaken. The plate was kept at room temperature for 20 min and absorbance was measured at a wavelength of 593 nm. The results are shown as µM Fe^2+^/g extract.

#### 4.5.4. Study of the Impact on the Formation of AOPP In Vitro by Moss Extracts

The study of the effect of water–ethanol extracts from four *Sphagnum* species was carried out using a modified method from Grzebyk and Piwowar [77]. First, a solution of BSA at a concentration of 40 mg/mL in PBS 0.01 M, pH 7.4 with chloramine T (20 mM) was prepared. The tested extracts dissolved in water at a concentration of 1 mg/mL were added to BSA solutions in the ratio 0.1/1.0 (*v/v*). All the samples were incubated for 60 min at 37 °C, which was followed by dialysis in PBS buffer for 24 h at 4 °C performed by introducing 5 changes of PBS buffer. The content of AOPP was determined spectrophotometrically according to the method described by Witko-Sarsat et al. [78]. The AOPP content in the samples is shown as µmol ChTE/mL. The solutions with the strongest protein inhibiting oxidation activity were determined to have the lowest AOPP content in the samples.

#### 4.5.5. Study of the Impact on PL by Moss Extracts

The study of the effect of extracts from four *Sphagnum* species on lipid peroxidation was carried out according to a modified method by Choochote et al. [79]. Extract samples (1 mg/mL) of 100 µL, 0.4 mL of 2.5% linoleic acid solution (*w*/*v* in 95% ethanol), 0.4 mL of PBS (10 mM, pH 7.4), and 0.1 mL of distilled water were added to Eppendorf tubes. All the ingredients were well mixed, and the Eppendorf tubes were left for 7 days in the dark at a temperature of 45 °C. The reaction with thiobarbituric acid was used to determine the amount of lipid peroxidation products formed. After 7 days, 0.5 mL of 20% trichloroacetic acid and 0.5 mL of 1% thiobarbituric acid in 0.05 N NaOH were added to 1 mL of the mixture. The contents of the tubes were heated in a boiling water bath for 20 min, then returned to room temperature and centrifuged at 4000 rpm for 15 min. The absorbance was measured at 532 nm and the % inhibition of peroxidation was calculated by the formula:

% inhibition of lipid peroxidation = [(Abs_control_ − Abs_sample_)/Abs_control_] × 100

where Abs_control_ is the absorbance of the sample solution mixture without the *Sphagnum* extract and Abs_sample_ is the absorbance of the mixture with the *Sphagnum* extract.

### 4.6. Study of the Influence of Moss Extracts on the Expression of Genes Encoding Selected Enzymes

#### 4.6.1. Cell Culture Conditions NHDF

Cells were cultured at 37 °C in a 5% CO_2_ incubator with the use of a FGM-2 Fibroblast Growth Medium-2 Bullet Kit containing FBM Basal Medium and FGM-2 SingleQuots supplements (human fibroblast growth factor-basic (hFGF-B), insulin and gentamicin). Cell numbers and their viability were monitored using a Countess TM Automated Cell Counter after 0.4% trypan blue staining.

The 3-[4,5-dimethylthiazol-2-yl]-2,5-diphenyltetrazolium bromide (MTT) assay was used to assess whether moss extracts SG, SM, SP and SS at concentrations between 15.6 μg/mL and 1000 μg/mL were toxic to NHDF cells. Appropriate concentrations of moss extracts were prepared in the culture medium. The NHDF cells were seeded into 96-well culture plates at a density of 1 × 10^4^ cells/well and were incubated with the moss extracts for 24 h. MTT (1.0 mg/mL) was added to the medium for 3 h before the end of the experiment. Next, the formazan crystals were dissolved in 100 μL of dimethyl sulfoxide and the absorbance was read at 540 nm using a microplate reader. Based on the MTT test results, moss extracts at a concentration of 125 μg/mL were selected for further research.

In the next step, the NHDF cells were seeded in 25 cm^2^ cell culture flasks and then lyophilized extracts were dissolved in culture medium; the appropriate amount was added to obtain a concentration of 125 μg/mL in each flask. Following 24 h of incubation, the cells were washed in PBS and pelleted for further molecular analyses. Untreated cells were used as a control. Each variant of the experiment was carried out in four biological repetitions.

#### 4.6.2. Molecular Analyses

The RNA extraction was performed using a TRIzol reagent according to the manufacturer’s protocols. The next stage of research included the qualitative and quantitative evaluation of RNA extracts with the use of agarose gel electrophoresis and a MaestroNano MN-913 spectrophotometer.

The mRNA levels of ELANE (elastase), HYAL1, HYAL2, HYLA3, HYAL4 (hyaluronidase 1–4), MMP1, MMP8, MMP13 (matrix metallopeptidase 1, 8 and 13), TYR (tyrosinase) and HAS1, HAS2, HAS3 (hyaluronan synthase 1–3) genes in cells exposed to moss extracts were determined using real-time RT-qPCR reactions. The gene expression levels were evaluated with SYBR Green I chemistry, and the RT-qPCR reactions were run on a LightCycler^®^ 480 Instrument II. The oligonucleotide specific primers used are commercially available. Each reaction was performed in duplicate. The thermal parameters for each reaction step were as follows: reverse transcription, 45 °C—10 min; activation, 95 °C—2 min, 45 cycles; denaturation, 95 °C—5 s; annealing, 60 °C—10 s; and extension, 72 °C—5 s. At the end, melting curve analysis was also performed.

The relative gene expression was determined based on the 2^−(ΔCt)^ method with normalization to GAPDH (glyceraldehyde-3-phosphate dehydrogenase), where ΔCt  =  Ct of the target—Ct of GAPDH [80].

### 4.7. Data Analysis

R language and environment, version 4.2.2 (R Core Team 2022) and libraries (stats, lme4, ade4, ggplot2, ggpubr, car, factoextra, lattice, and MuMIn) were used for all statistical analyses and to visualize the results. To examine the impact of the extracts of the four moss species (SG, SM, SP and SS) and the content of phenols (TP), phenolic acids (TPA) and flavonoids (TF) on antioxidant properties (ABTS, DPPH, FRAP), PL and creation of AOPP, Linear Mixed-Effects Models (LMM) with Gaussian distribution were applied. This procedure combines analysis of the impact of the factor variable (moss effect in our case) and continuous variables (TP, TPA, TF). In LMM, the effects of continuous variables were treated as fixed (under control) variables, while the species identity of *Sphagnum* spp. (SG, SM, SP and SS) was regarded as a random variable. Due to the relatively small dataset, it was not possible to carry out correlation analysis among the variables for each species separately. Thus, we applied the effect of moss as a random variable. We assumed that the effect within specific taxon would be more similar than among taxa. The selection of the best-fit models of the LMM was performed using the function dredge in the package “MuMIn”. This procedure indicates models with the lowest corrected Akaike information (AICc) and the lowest delta (D). Apart from coefficients of the model (full and conditional), the significance levels of the final model based on Wald chi-square statistics and *p*-values of each covariate are presented.

The overall differences in expression of genes coding some enzymes (ELANE, HYAL1, HYAL2, HYAL3, HYAL4, MMP1, MMP8, MMP13, TYR, HAS1, HAS2, HAS3) in fibroblasts of human skin among groups (control and particular mosses) were tested using Principal Components Analysis (PCA). The contributions of particular variables were identified and the significance of differences (variance partitioning) were calculated using the Monte Carlo test (999 iterations). The differences in expression of particular genes coding some enzymes in fibroblasts of human skin among groups (control and particular mosses) were tested using analysis of variance (ANOVA) and LSD Fisher test for pairwise comparisons when ANOVA yielded a significant result. The distribution of normality was checked by the Shapiro–Wilk test. The same LMM was applied to examine the effect of extracts of the four moss species (SG, SM, SP and SS) and content of phenols (TP), phenolic acids (TPA) and flavonoids (TF) on the expression of genes coding the aforementioned enzymes.

In order to visualize the results of the LMM, barplots showing the effects of moss and scatterplots showing the effects of the continuous variables on antioxidant properties and enzymes are presented.

## 5. Conclusions

In conclusion, this paper presents the antioxidant properties of water–ethanol extracts obtained from *Sphagnum girgensohnii*, *S. magellanicum, S. palustre*, *S. squarrosum*. What is worth emphasizing is that this is the first in vitro study of the effect of extracts from plants of the genus *Sphagnum* on the expression of genes encoding enzymes related to the condition of the skin. Despite the different content of phenolic compounds, the extracts from all *Sphagnum* species which were tested had a similar effect on the expression of the genes encoding the studied enzymes. Assuming that the modulation of gene expression is responsible for the reduced or increased synthesis of the tested enzymes, it can be concluded, based on the obtained results, that the potential use of the tested extracts from various *Sphagnum* species in cosmetics aimed at reducing the signs of skin aging would not be a beneficial solution. This conclusion is based on the possibility of accelerating the degradation of collagen and hyaluronic acid and inhibiting the synthesis of hyaluronic acid. Based on the results of this work, we cannot confirm the hypothesis stated earlier that extracts from *Sphagnum* species could be effective components of anti-aging preparations, despite their antioxidant activity. The obtained results suggest the need for further research on the effects of *Sphagnum* extracts.

## Figures and Tables

**Table 1:** The content of selected phenolic compounds determined by HPLC in *Sphagnum* extracts.

Species of Moss	Content of Determined Phenolic Compounds in mg/g of Dry Extract			
	*p*-Coumaric Acid	Rutin	Quercetin	Apigenin
*Sphagnum girgensohnii* Russow.	0.141	0.064	-	-
*Sphagnum magellanicum* Brid.	0.069	0.510	0.142	-
*Sphagnum palustre* L.	0.104	0.364	-	-
*Sphagnum squarrosum* Crome	0.263	0.102	-	0.053

For the standard error, see Appendix A Table A1. “-” indicates that a specific compound concentration was under the detection limit.

**Table 2:** Description of morphological features and habitat preferences of the studied species from the genus *Sphagnum*.

Moss Species Name	Photographs of Species	Morphological Features	Habitats
*Sphagnum girgensohnii* Russow.	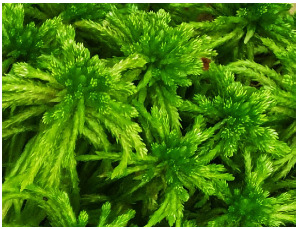	Rather robust, green or yellowish-green plants, forming loose hummocks or mats	Wet forests and thicket communities, mid-forest peat bogs,
*Sphagnum magellanicum* Brid.	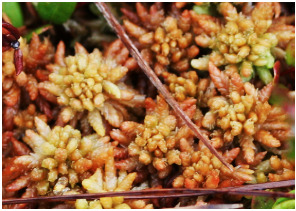	Robust plants, most often reddish to wine-red in colour, forming broad carpets or hummocks	Oligotrophic, most often raised and transitional bogs, marshy forests and birch forests, less often acidic low bogs
*Sphagnum palustre* L.	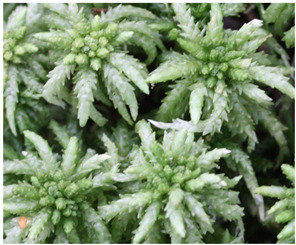	Robust plants, usually green, pale green, yellow-green, sometimes orange or brownish, forming loose carpets or tussocks	Swamp forests, low, transitional and raised bogs
*Sphagnum squarrosum* Crome	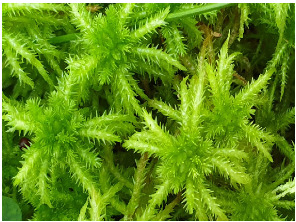	Large plants, with characteristically strongly bent branch leaves, from light green to yellow-green, in sunny places sometimes brownish, forming loose mats	Wet forests, thickets, stream banks, mid-forest springs and banks of water reservoirs

## Data Availability

Data is contained within the article and Supplementary Material.

## Supplementary Materials

The following supporting information can be downloaded at: https://www.mdpi.com/article/10.3390/ph16081076/s1, Table S1: The results of Linear Mixed Effects Models including impact of moss species identity, total phenols content (TP), total phenolic acids content (TPA) and total flavonoids content (TF) on antioxidant properties; Table S2: The results of Linear Mixed Effects Models including impact of moss species identity, total phenols content (TP), total phenolic acids content (TPA) and total flavonoids content (TF) on enzymes genes expression. Figure S1: Chromatogram of *Sphagnum girgensohnii* Russow.; Figure S2: Chromatogram of *Sphagnum magellanicum* Brid.; Figure S3: Chromatogram of *Sphagnum palustre* L.; Figure S4: Chromatogram of *Sphagnum squarrosum* Crome.

## Abbreviations

AOPP—advanced oxidation protein products; BSA—bovine serum albumin; CAE—caffeic acid equivalents; ChTE—chloramine T equivalents; ECM—extracellular matrix; Fe^2+^E—Fe^2+^ equivalents; GAE—gallic acid equivalents; HA—hyaluronic acid; LMM—Linear Mixed-Effects Models; MMPs—matrix metaloproteinases; NHDF—normal human dermal fibroblasts; PCA—Principal Components Analysis; PBS—phosphate buffered saline; PL—peroxidation of lipids; QE—quercetin equivalents; SG—*Sphagnum girgensohnii*; SM—*Sphagnum magellanicum*; SP—*Sphagnum palustre*; SS—*Sphagnum squarrosum*; TE—trolox equivalents; TF—total flavonoids content; TP—total phenols content; TPA—total phenolic acids content.

## Appendix A

**Table A1:** Validation data for the determination of phenolic compounds by HPLC.

Parameter	Phenolic Compounds			
	*p*-Coumaric Acid	Rutin	Quercetin	Apigenin
LOD (mg/L)	0.25	0.32	0.21	0.12
LOQ (mg/L)	0.83	1.05	0.70	0.38
Standard error (mg/L)	0.08	0.10	0.07	0.04
Linearity range (mg/L)	0.83–100	1.05–100	0.70–100	0.38–100

## References

[1] Oke T.A., Hager H.A. (2020). Plant community dynamics and carbon sequestration in *Sphagnum*-dominated peatlands in the era of global change. Glob. Ecol. Biogeogr..

[2] Shaw A.J., Cox C.J., Buck W.R., Devos N., Buchanan A.M., Cave L., Seppelt R., Shaw B., Larraín J., Andrus R. (2010). Newly resolved relationships in an early land plant lineage: Bryophyta class *Sphagnopsida* (peat mosses). Am. J. Bot..

[3] Hodgetts N.G., Söderström L., Blockeel T.L., Caspari S., Ignatov M.S., Konstantinova N.A., Lockhart N., Papp B., Schröck C., Sim-Sim M. (2020). An annotated checklist of bryophytes of Europe, Macaronesia and Cyprus. J. Bryol..

[4] Stebel A. (2017). Peat Mosses of the ‘Lasy Środkowopomorskie’ Promotional Forest Complex (Poland, West Pomerania).

[5] Rasmussen S., Wolff C., Rudolph H. (1995). Compartmentalization of phenolic constituents in *Sphagnum*. Phytochemistry.

[6] Stalheim T., Ballance S., Christensen B.E., Granum P.E. (2009). Sphagnan—A pectin-like polymer isolated from *Sphagnum* moss can inhibit the growth of some typical food spoilage and food poisoning bacteria by lowering the pH. J. Appl. Microbiol..

[7] Painter T.J., Christensen B.E. (2003). Concerning the wound-healing properties of *Sphagnum* holocellulose: The Maillard reaction in pharmacology. J. Ethnopharmacol..

[8] Klavina L., Springe G., Nikolajeva V., Martsinkevich I., Nakurte I., Dzabijeva D., Steinberga I. (2015). Chemical composition analysis, antimicrobial activity and cytotoxicity screening of moss extracts (Moss Phytochemistry). Molecules.

[9] Kang H.R., Lee D., Eom H.J., Lee S.R., Lee K.R., Kang K.S., Kim K.H. (2016). Identification and mechanism of action of renoprotective constituents from peat moss *Sphagnum palustre* in cisplatin-induced nephrotoxicity. J. Funct. Foods.

[10] Nam J.-H., Jeong J.-C., Yoon Y.-H., Hong S.-Y., Kim S.-J., Jin Y.-I., Lee Y.-J., Yoo D.-L., Lee K.-T., Park H.-J. (2011). Phytochemical constituents and anticancer activity of *Sphagnum palustre* extract. Korean J. Plant Resour..

[11] Montenegro G., Portaluppi M.C., Salas F.A., Díaz M.F. (2009). Biological properties of the Chilean native moss *Sphagnum magellanicum*. Biol. Res..

[12] Eom H.J., Park Y.J., Kang H.R., Kim H.R., Bang I.J., Park H.B., Chung K.H., Kim K.H. (2016). Inhibitory effect of *Sphagnum palustre* extract and its bioactive compounds on aromatase activity. Bangladesh J. Pharmacol..

[13] Mellegård H., Stalheim T., Hormazabal V., Granum P.E., Hardy S.P. (2009). Antibacterial activity of sphagnum acid and other phenolic compounds found in *Sphagnum papillosum* against food-borne bacteria. Lett. Appl. Microbiol..

[14] Fudyma J.D., Lyon J., AminiTabrizi R., Gieschen H., Chu R.K., Hoyt D.W., Kyle J.E., Toyoda J., Tolic N., Heyman H.M. (2019). Untargeted metabolomic profiling of *Sphagnum fallax* reveals novel antimicrobial metabolites. Plant Direct.

[15] Tienaho J., Silvan N., Muilu-Mäkelä R., Kilpeläinen P., Poikulainen E., Sarjala T. (2021). Ultraviolet absorbance of Sphagnum magellanicum, S. fallax and S. fuscum extracts with seasonal and species-specific variation. Photochem. Photobiol. Sci..

[16] Mejía-Giraldo J.C., Gallardo C., Puertas-Mejía M.A. (2015). In vitro photoprotection and antioxidant capacity of *Sphagnum meridense* extracts, a novel source of natural sunscreen from the mountains of Colombia. Pure Appl. Chem..

[17] Joshi S., Singh S., Sharma R., Vats S., Nagaraju G.P., Alam A. (2022). Phytochemical screening and antioxidant potential of *Plagiochasma appendiculatum* Lehm. & Lindenb. and *Sphagnum fimbriatum* Wilson. Plant Sci. Today.

[18] Benek A., Canli K., Altuner E.M. (2022). Traditional medicinal uses of mosses. Anatol. Bryol..

[19] Drobnik J., Stebel A. (2017). Tangled history of the European uses of *Sphagnum* moss and sphagnol. J. Ethnopharmacol..

[20] Opelt K., Berg C., Berg G. (2007). The bryophyte genus *Sphagnum* is a reservoir for powerful and extraordinary antagonists and potentially facultative human pathogens. FEMS Microbiol. Ecol..

[21] Dziwak M., Wróblewska K., Szumny A., Galek R. (2022). Modern use of Bryophytes as a source of secondary metabolites. Agronomy.

[22] Podterob A.P., Zubets E.V. (2002). A history of the medicinal use of plants of the genus *Sphagnum*. Pharm. Chem. J..

[23] Zillich O.V., Schweiggert-Weisz U., Eisner P., Kerscher M. (2015). Polyphenols as active ingredients for cosmetic products. Int. J. Cosmet. Sci..

[24] Ferreira M.S., Magalhães M.C., Oliveira R., Sousa-Lobo J.M., Almeida I.F. (2021). Trends in the use of botanicals in anti-aging cosmetics. Molecules.

[25] Thring T.S.A., Hili P., Naughton D.P. (2009). Anti-collagenase, anti-elastase and anti-oxidant activities of extracts from 21 plants. BMC Complement. Altern. Med..

[26] Bazzicalupo M., Burlando B., Denaro M., Barreca D., Trombetta D., Smeriglio A., Cornara L. (2019). Polyphenol characterization and skin-preserving properties of hydroalcoholic flower extract from *Himantoglossum robertianum* (Orchidaceae). Plants.

[27] Marques R.V., Guillaumin A., Abdelwahab A.B., Salwinski A., Gotfredsen C.H., Bourgaud F., Enemark-rasmussen K., Miguel S., Simonsen H.T. (2021). Collagenase and tyrosinase inhibitory effect of isolated constituents from the moss *Polytrichum formosum*. Plants.

[28] Chiocchio I., Mandrone M., Sanna C., Maxia A., Tacchini M., Poli F. (2018). Screening of a hundred plant extracts as tyrosinase and elastase inhibitors, two enzymatic targets of cosmetic interest. Ind. Crops Prod..

[29] Andrade J.M., Domínguez-Martín E.M., Nicolai M., Faustino C., Rodrigues L.M., Rijo P. (2021). Screening the dermatological potential of *Plectranthus* species components: Antioxidant and inhibitory capacities over elastase, collagenase and tyrosinase. J. Enzym. Inhib. Med. Chem..

[30] Jiratchayamaethasakul C., Ding Y., Hwang O., Im S.T., Jang Y., Myung S.W., Lee J.M., Kim H.S., Ko S.C., Lee S.H. (2020). In vitro screening of elastase, collagenase, hyaluronidase, and tyrosinase inhibitory and antioxidant activities of 22 halophyte plant extracts for novel cosmeceuticals. Fish. Aquat. Sci..

[31] Motti R., Di Palma A., de Falco B. (2023). Bryophytes used in folk medicine: An ethnobotanical overview. Horticulturae.

[32] Bandyopadhyay A., Dey A. (2022). The ethno-medicinal and pharmaceutical attributes of Bryophytes: A review. Phytomed. Plus.

[33] Chandra S., Chandra D., Barh A., Pankaj, Pandey R.K., Sharma I.P. (2017). Bryophytes: Hoard of remedies, an ethno-medicinal review. J. Tradit. Complement. Med..

[34] Niederkrüger H., Busch A., Dabrowska-Schlepp P., Krieghoff N., Schaaf A., Frischmuth T. (2019). Single-use processing as a safe and convenient way to develop and manufacture moss-derived biopharmaceuticals. Single-Use Technology in Biopharmaceutical Manufacture.

[35] Decker E.L., Reski R. (2020). Mosses in biotechnology. Curr. Opin. Biotechnol..

[36] Arda O., Göksügür N., Tüzün Y. (2014). Basic histological structure and functions of facial skin. Clin. Dermatol..

[37] Pittayapruek P., Meephansan J., Prapapan O., Komine M., Ohtsuki M. (2016). Role of matrix metalloproteinases in photoaging and photocarcinogenesis. Int. J. Mol. Sci..

[38] Shin J.W., Kwon S.H., Choi J.Y., Na J.I., Huh C.H., Choi H.R., Park K.C. (2019). Molecular mechanisms of dermal aging and anti-aging approaches. Int. J. Mol. Sci..

[39] Zhang G., Wang P., Wang X. (2017). Skin ageing and cancer. The Role of Matrix Metalloproteinase in Human Body Pathologies.

[40] Chen J., Liu Y., Zhao Z., Qiu J. (2021). Oxidative stress in the skin: Impact and related protection. Int. J. Cosmet. Sci..

[41] De Lima Cherubim D.J., Buzanello Martins C.V., Oliveira Fariña L., da Silva de Lucca R.A. (2020). Polyphenols as natural antioxidants in cosmetics applications. J. Cosmet. Dermatol..

[42] Smolińska-Kondla D., Zych M., Ramos P., Wacławek S., Stebel A. (2022). Antioxidant potential of various extracts from 5 common European mosses and its correlation with phenolic compounds. Herba Pol..

[43] Klavina L., Springe G. (2015). Optimisation of conditions for extraction of biologically active compounds from common Bryophytes in Latvia. Proc. Latv. Acad. Sci. Sect. B Nat. Exact Appl. Sci..

[44] Piwowar A., Rorbach-Dolata A., Fecka I. (2019). The antiglycoxidative ability of selected phenolic compounds—An in vitro study. Molecules.

[45] Laoung-On J., Jaikang C., Saenphet K., Sudwan P. (2021). Phytochemical screening, antioxidant and sperm viability of *Nelumbo nucifera* petal extracts. Plants.

[46] Martínez S., Fuentes C., Carballo J. (2022). Antioxidant activity, total phenolic content and total flavonoid content in sweet chestnut (*Castanea sativa* Mill.) cultivars grown in Northwest Spain under different environmental conditions. Foods.

[47] Kumar D., Ladaniya M.S., Gurjar M., Kumar S. (2022). Impact of drying methods on natural antioxidants, phenols and flavanones of immature dropped *Citrus sinensis* L. Osbeck fruits. Sci. Rep..

[48] Chew K.K., Khoo M.Z., Ng S.Y., Thoo Y.Y., Aida W.W., Ho C.W. (2011). Effect of ethanol concentration, extraction time and extraction temperature on the recovery of phenolic compounds and antioxidant capacity of *Orthosiphon stamineus* extracts. Int. Food Res. J..

[49] Thoo Y.Y., Ho K.S., Liang Y.J., Ho C.W., Tan P.C. (2010). Effects of binary solvent extraction system, extraction time and extraction temperature on phenolic antioxidants and antioxidant capacity from mengkudu (*Morinda citrifolia*). Food Chem..

[50] Wolski G.J., Sadowska B., Fol M., Podsędek A., Kajszczak D., Kobylińska A. (2021). Cytotoxicity, antimicrobial and antioxidant activities of mosses obtained from open habitats. PLoS ONE.

[51] Villarroel M., Acevedo C., Yáñez E., Biolley E. (2003). Functional properties of *Sphagnum magellanicum* fiber and its direct use in formulation of bakery products. Arch. Latinoam. Nutr..

[52] Chang T.S. (2009). An updated review of tyrosinase inhibitors. Int. J. Mol. Sci..

[53] Zolghadri S., Bahrami A., Hassan Khan M.T., Munoz-Munoz J., Garcia-Molina F., Garcia-Canovas F., Saboury A.A. (2019). A comprehensive review on tyrosinase inhibitors. J. Enzyme Inhib. Med. Chem..

[54] Lunić T.M., Oalde M.M., Mandić M.R., Sabovljević A.D., Sabovljević M.S., Gašić U.M., Duletić-Laušević S.N., Božić B.D., Nedeljković B.D.B. (2020). Extracts characterization and in vitro evaluation of potential immunomodulatory activities of the moss *Hypnum cupressiforme* Hedw. Molecules.

[55] Fan M., Zhang G., Hu X., Xu X., Gong D. (2017). Quercetin as a tyrosinase inhibitor: Inhibitory activity, conformational change and mechanism. Food Res. Int..

[56] Gasowska-Bajger B., Wojtasek H. (2016). Reactions of flavonoids with o-quinones interfere with the spectrophotometric assay of tyrosinase activity. J. Agric. Food Chem..

[57] Wojtasek H. (2022). Comment on “Natural and synthetic flavonoid derivatives as new potential tyrosinase inhibitors: A systematic review” by R. Obaid, E. Mughal, N. Naeem, A. Sadiq, R.; Alsantali, R. Jassas, Z. Moussa and S. Ahmed, RSC Advances, **2021**, *11*, 22159. RSC Adv..

[58] Promden W., Viriyabancha W., Monthakantirat O., Umehara K., Noguchi H., De-Eknamkul W. (2018). Correlation between the potency of flavonoids on mushroom tyrosinase inhibitory activity and melanin synthesis in melanocytes. Molecules.

[59] Park K.Y., Kim J. (2020). Synthesis and biological evaluation of the anti-melanogenesis effect of coumaric and caffeic acid-conjugated peptides in human melanocytes. Front. Pharmacol..

[60] Tardif G., Reboul P., Pelletier J.-P., Martel-Pelletier J. (2004). Ten years in the life of an enzyme: The story of the human MMP-13 (collagenase-3). Mod. Rheumatol..

[61] Philips N., Auler S., Hugo R., Gonzalez S. (2011). Beneficial regulation of matrix metalloproteinases for skin health. Enzyme Res..

[62] Azmi N., Hashim P., Hashim D.M., Halimoon N., Nik Majid N.M. (2014). Anti-elastase, anti-tyrosinase and matrix metalloproteinase-1 inhibitory activity of earthworm extracts as potential new anti-aging agent. Asian Pac. J. Trop. Biomed..

[63] Heinz A. (2020). Elastases and elastokines: Elastin degradation and its significance in health and disease. Crit. Rev. Biochem. Mol. Biol..

[64] Avantaggiato A., Bertuzzi G., Vitiello U., Iannucci G., Pasin M., Pascali M., Cervelli V., Carinci F. (2014). Role of antioxidants in dermal aging: An in vitro study by q-RT-PCR. Aesthetic Plast. Surg..

[65] Tsukahara K., Takema Y., Moriwaki S., Tsuji N., Suzuki Y., Fujimura T., Imokawa G. (2001). Selective inhibition of skin fibroblast elastase elicits a concentration-dependent prevention of ultraviolet B-induced wrinkle formation. J. Investig. Dermatol..

[66] Tjermin L., Nyoman I., Lister E., Nasution A.N., Girsang E. (2019). Antioxidant and inhibition of elastase effect of scutellarein and apigenin. Technol. Sci. Am. Sci. Res. J. Eng..

[67] Kavasi R.M., Berdiaki A., Spyridaki I., Corsini E., Tsatsakis A., Tzanakakis G., Nikitovic D. (2017). HA metabolism in skin homeostasis and inflammatory disease. Food Chem. Toxicol..

[68] Cavallini M., Gazzola R., Metalla M., Vaienti L. (2013). The role of hyaluronidase in the treatment of complications from hyaluronic acid dermal fillers. Aesthetic Surg. J..

[69] Fisher G.J., Wang B., Cui Y., Shi M., Zhao Y., Quan T., Voorhees J.J. (2023). Skin aging from the perspective of dermal fibroblasts: The interplay between the adaptation to the extracellular matrix microenvironment and cell autonomous processes. J. Cell Commun. Signal..

[70] Qin Z., Balimunkwe R.M., Quan T. (2017). Age-related reduction of dermal fibroblast size upregulates multiple matrix metalloproteinases as observed in aged human skin in vivo. Br. J. Dermatol..

[71] Oh J.-H., Kim Y.K., Jung J.-Y., Shin J.-E., Kim K.H., Cho K.H., Eun H.C., Chung J.H. (2011). Intrinsic aging- and photoaging-dependent level changes of glycosaminoglycans and their correlation with water content in human skin. J. Dermatol. Sci..

[72] Mihailović V., Kreft S., Benković E.T., Ivanović N., Stanković M.S. (2016). Chemical profile, antioxidant activity and stability in stimulated gastrointestinal tract model system of three *Verbascum* species. Ind. Crops Prod..

[73] Knoll J.E. (1985). Estimation of the limit of detection in chromatography. J. Chromatogr. Sci..

[74] Bobo-García G., Davidov-Pardo G., Arroqui C., Vírseda P., Marín-Arroyo M.R., Navarro M. (2015). Intra-laboratory validation of microplate methods for total phenolic content and antioxidant activity on polyphenolic extracts, and comparison with conventional spectrophotometric methods. J. Sci. Food Agric..

[75] Re R., Pellegrini N., Proteggente A., Pannala A., Yang M., Rice-Evans C. (1999). Antioxidant activity applying an improved ABTS radical cation decolorization assay. Free Radic. Biol. Med..

[76] Benzie I.F.F., Strain J.J. (1996). The ferric reducing ability of plasma (FRAP) as a measure of “antioxidant power”: The FRAP assay. Anal. Biochem..

[77] Grzebyk E., Piwowar A. (2014). The Tibetan herbal medicines Padma 28 and Padma Circosan inhibit the formation of advanced glycation endproducts (AGE) and advanced oxidation protein products (AOPP) in vitro. BMC Complement. Altern. Med..

[78] Witko-Sarsat V., Friedlander M., Capeillère-Blandin C., Nguyen-Khoa T., Nguyen A.T., Zingraff J., Jungers P., Descamps-Latscha B. (1996). Advanced oxidation protein products as a novel marker of oxidative stress in uremia. Kidney Int..

[79] Choochote W., Suklampoo L., Ochaikul D. (2014). Evaluation of antioxidant capacities of green microalgae. J. Appl. Phycol..

[80] Schmittgen T.D., Livak K.J. (2008). Analyzing real-time PCR data by the comparative CT method. Nat. Protoc..

