# Supplementary material for: Effects of Water–Ethanol Extracts from Four Sphagnum Species on Gene Expression of Selected Enzymes in Normal Human Dermal Fibroblasts and Their Antioxidant Properties
Source: Pharmaceuticals (Basel). 2023 Jul 28;16(8):1076. doi: 10.3390/ph16081076 (PMC10458669; doi:10.3390/ph16081076)
Supplement: Supplementary file 1 [file pharmaceuticals-16-01076-s001.zip › pharmaceuticals-2410174-supplementary.pdf]

pharmaceuticals

MDPI

Supplementary materials

Table S1. The results of Linear Mixed Effects Models including impact of moss species identity, total phenols content (TP), total phenolic acids content (TPA) and total flavonoids content (TF) on antioxidant properties. The significant p-values were bolded.

Variable	Estimate	SE	Adj SE	z	P-value	Variable	Chisq	Df	P-value
	**ABTS** full average coefficients								
Intercept	257.6591	44.93292	48.23132	5.342	**<0.0001**	TP	0.1701	1	0.68
SM	25.48165	9.87407	10.49916	2.427	**0.015223**	TPA	1.7386	1	0.1873
SP	53.98851	8.23326	8.84245	6.106	**<0.0001**	TF	21.2814	1	**<0.0001**
SS	-46.0785	11.51261	12.3309	3.737	**0.000186**	moss	86.0089	3	**<0.0001**
TF	-19.7933	4.18129	4.47695	4.421	**<0.0001**				
TPA	0.22942	0.65228	0.67553	0.34	0.73415				
TP	0.03451	0.44238	0.47488	0.073	0.942065				
	conditional average coefficients								
Intercept	257.6591	44.9329	48.2313	5.342	**<0.0001**				
SM	25.4944	9.8601	10.4863	2.431	**0.015048**				
SP	54.0155	8.1462	8.7618	6.165	**<0.0001**				
SS	-46.1016	11.4693	12.2908	3.751	**0.000176**				
TF	-19.8331	4.0901	4.3924	4.515	**<0.0001**				
TPA	1.2896	1.0121	1.0945	1.178	0.238703				
TP	0.3782	1.4194	1.5302	0.247	0.804771				
	**DPPH** full average coefficients								
Intercept	53.24122	30.81867	31.60734	1.684	0.0921	TP	0.001	1	0.9748
SM	34.81441	6.60436	6.8218	5.103	**<0.0001**	TPA	0.3204	1	0.57135
SP	12.16988	5.64893	5.84136	2.083	**0.0372**	TF	3.2557	1	0.07118
SS	-11.6317	7.77383	7.97946	1.458	0.1449	moss	178.712	3	**<0.0001**
TF	-2.23689	2.81602	2.88403	0.776	0.438				
TP	-0.09069	0.37627	0.39264	0.231	0.8173				
TPA	0.00216	0.21485	0.22872	0.009	0.9925				
	conditional average coefficients								
Intercept	53.24122	30.81867	31.60734	1.684	0.0921				
SM	34.83942	6.54044	6.76009	5.154	**<0.0001**				
SP	12.17863	5.64152	5.83434	2.087	**0.0369**				
SS	-11.6401	7.77036	7.97623	1.459	0.1445				
TF	-4.70045	2.25469	2.42864	1.935	0.0529				
TP	-0.60861	0.79681	0.84817	0.718	0.473				
TPA	0.01934	0.64262	0.68413	0.028	0.9775				
	**FRAP** full average coefficients								
Intercept	44.83117	24.89747	25.60028	1.751	0.079912	TP	8.5871	1	**0.00339**
SM	49.63696	13.08715	13.29593	3.733	**0.000189**	TPA	0.9252	1	0.33612
SP	15.98666	4.22915	4.44404	3.597	**0.000322**	TF	8.1576	1	**0.00429**
SS	-5.47168	9.56442	9.73321	0.562	0.574003	moss	323.998	3	**<0.0001**
TF	2.11798	3.02768	3.08309	0.687	0.492104				
TP	-0.69851	0.92941	0.94622	0.738	0.460384				
TPA	-0.01696	0.19017	0.20217	0.084	0.933162				
	conditional average coefficients								
Intercept	44.8312	24.8975	25.6003	1.751	0.079912				
SM	49.6487	13.0663	13.2755	3.74	**0.000184**				
SP	15.9905	4.2225	4.4377	3.603	**0.000314**				

SS	-5.473	9.5652	9.734	0.562	0.573943				
TF	5.0284	2.6699	2.8165	1.785	0.074199				
TP	-1.5318	0.786	0.8288	1.848	0.06457				
TPA	-0.1576	0.5604	0.5982	0.264	0.792152				
**PL** full average coefficients									
Intercept	7.0948	5.26581	5.44906	1.302	0.193	TP	0.1132	1	0.7365
TF	0.56014	0.54409	0.55589	1.008	0.314	TPA	0.9634	1	0.3263
TPA	-0.26905	0.28412	0.29043	0.926	0.354	TF	0.002	1	0.9644
SM	-0.51516	1.48975	1.53211	0.336	0.737	moss	2.45	3	0.4844
SP	0.36824	1.12058	1.15807	0.318	0.75				
SS	-0.24169	0.95694	1.00128	0.241	0.809				
TP	-0.03565	0.13094	0.13623	0.262	0.794				
conditional average coefficients									
Intercept	7.0948	5.2658	5.4491	1.302	0.1929				
TF	0.815	0.4723	0.4919	1.657	0.0975				
TPA	-0.4696	0.2161	0.2303	2.039	0.0414				
SM	-2.8374	2.3737	2.5179	1.127	0.2598				
SP	2.0283	1.884	2.0049	1.012	0.3117				
SS	-1.3312	1.8956	2.0178	0.66	0.5094				
TP	-0.1473	0.2332	0.2455	0.6	0.5483				

Table S2. The results of Linear Mixed Effects Models including impact of moss species identity, total phenols content (TP), total phenolic acids content (TPA) and total flavonoids content (TF) on enzymes genes expression. The significant p-values were bolded.

Variable	Estimate	SE	Adj SE	z	P-value		Chisq	Df	Pr(>Chisq)
ELANE (full average coefficients)									
(Intercept)	3.160e-05	2.098e-05	2.165e-05	1.460	0.144	TP	0.004	1	0.94979
mossSM	4.665e-06	8.490e-06	8.640e-06	0.540	0.589	TPA	18.1259	1	**0.00002068**
mossSP	-2.365e-07	3.826e-06	4.000e-06	0.059	0.953	TF	2.1654	1	0.14115
mossSS	-4.393e-07	5.209e-06	5.372e-06	0.082	0.935	moss	15.6464	3	0.00134
TPA	-1.837e-06	1.408e-06	1.431e-06	1.284	0.199				
TP	2.278e-07	5.573e-07	5.721e-07	0.398	0.690				
TF	5.389e-07	2.072e-06	2.122e-06	0.254	0.800				
(conditional average coefficients)									
(Intercept)	3.160e-05	2.098e-05	2.165e-05	1.460	0.1444				
mossSM	1.351e-05	9.447e-06	9.834e-06	1.374	0.1694				
mossSP	-6.852e-07	6.489e-06	6.785e-06	0.101	0.9196				
mossSS	-1.273e-06	8.806e-06	9.085e-06	0.140	0.8886				
TPA	-2.351e-06	1.153e-06	1.188e-06	1.979	**0.0479**				
TP	7.498e-07	7.941e-07	8.282e-07	0.905	0.3653				
TF	2.574e-06	3.908e-06	4.034e-06	0.638	0.5235				
HYAL1 (full average coefficients)									
(Intercept)	2.779e-05	5.433e-06	5.675e-06	4.897	**0.000001**	TP	0.8812	1	0.348
TPA	-8.572e-07	6.882e-07	7.040e-07	1.218	0.223	TPA	7.6769	1	**0.006**
TP	1.283e-07	3.447e-07	3.536e-07	0.363	0.717	TF	0.6552	1	0.418

TF	-7.577e-08	3.173e-07	3.325e-07	0.228	0.820	moss	2.6793	3	0.444
mossSM	1.182e-07	1.257e-06	1.288e-06	0.092	0.927				
mossSP	6.688e-08	7.744e-07	8.015e-07	0.083	0.934				
mossSS	3.045e-08	5.757e-07	6.082e-07	0.050	0.960				
(conditional average coefficients)									
(Intercept)	2.779e-05	5.433e-06	5.675e-06	4.897	**0.000001**				
TPA	-1.092e-06	5.892e-07	6.126e-07	1.782	0.0747				
TP	3.702e-07	5.032e-07	5.208e-07	0.711	0.4771				
TF	-3.668e-07	6.170e-07	6.546e-07	0.560	0.5752				
mossSM	6.334e-06	6.732e-06	7.032e-06	0.901	0.3677				
mossSP	3.583e-06	4.420e-06	4.671e-06	0.767	0.4430				
mossSS	1.631e-06	3.892e-06	4.148e-06	0.393	0.6941				
HYAL2 (full average coefficients)									
(Intercept)	2.220e-03	6.573e-04	6.751e-04	3.288	**0.00101**	TP	0.0429	1	0.835828
mossSM	1.910e-04	3.466e-04	3.508e-04	0.545	0.58601	TPA	9.0561	1	**0.002618**
mossSP	7.880e-05	1.659e-04	1.706e-04	0.462	0.64418	TF	0.0093	1	0.92297
mossSS	-3.526e-05	1.141e-04	1.201e-04	0.294	0.76906	moss	10.1493	3	**0.017338**
TPA	-4.254e-05	5.650e-05	5.724e-05	0.743	0.45734				
TP	1.180e-05	2.382e-05	2.432e-05	0.485	0.62747				
TF	-3.571e-06	3.342e-05	3.545e-05	0.101	0.91976				
(conditional average coefficients)									
(Intercept)	2.220e-03	6.573e-04	6.751e-04	3.288	**0.00101**				
mossSM	6.856e-04	3.036e-04	3.202e-04	2.141	**0.03224**				
mossSP	2.828e-04	2.028e-04	2.163e-04	1.308	0.19104				
mossSS	-1.265e-04	1.877e-04	2.005e-04	0.631	0.52802				
TPA	-8.456e-05	5.284e-05	5.440e-05	1.554	0.12007				
TP	3.358e-05	2.972e-05	3.085e-05	1.088	0.27647				
TF	-2.058e-05	7.803e-05	8.302e-05	0.248	0.80419				
HYAL3 (full average coefficients)									
(Intercept)	3.418e-06	1.976e-06	2.054e-06	1.664	0.0961	TP	1.7068	1	0.191
TF	2.896e-08	1.345e-07	1.405e-07	0.206	0.8367	TPA	3.9951	1	**0.046**
TP	1.584e-08	7.372e-08	7.622e-08	0.208	0.8354	TF	0.2783	1	0.598
TPA	-1.336e-08	1.050e-07	1.086e-07	0.123	0.9021	moss	6.3106	3	0.097
mossSM	7.298e-08	6.388e-07	6.525e-07	0.112	0.9109				
mossSP	6.821e-08	5.025e-07	5.123e-07	0.133	0.8941				
mossSS	-1.537e-08	2.959e-07	3.103e-07	0.050	0.9605				
(conditional average coefficients)									
(Intercept)	3.418e-06	1.976e-06	2.054e-06	1.664	0.0961				
TF	1.244e-07	2.566e-07	2.701e-07	0.461	0.6451				
TP	6.639e-08	1.394e-07	1.449e-07	0.458	0.6469				
TPA	-6.544e-08	2.250e-07	2.332e-07	0.281	0.7790				
mossSM	2.375e-06	2.795e-06	2.896e-06	0.820	0.4122				

mossSP	2.220e-06	1.855e-06	1.941e-06	1.144	0.2527				
mossSS	-5.003e-07	1.614e-06	1.700e-06	0.294	0.7686				
HYAL4 (full average coefficients)									
(Intercept)	7.222e-05	7.120e-05	7.187e-05	1.005	0.315	TP	0.2229	1	0.6368498
mossSM	-6.592e-06	1.197e-05	1.215e-05	0.542	0.588	TPA	1.0259	1	0.3111236
mossSP	7.750e-06	1.137e-05	1.152e-05	0.673	0.501	TF	4.0077	1	**0.0452926**
mossSS	-1.324e-05	1.903e-05	1.919e-05	0.690	0.490	moss	16.44	3	**0.0009211**
TF	-3.914e-06	6.248e-06	6.309e-06	0.620	0.535				
TP	-1.010e-07	4.658e-07	4.834e-07	0.209	0.835				
TPA	-1.623e-07	5.235e-07	5.411e-07	0.300	0.764				
(conditional average coefficients)									
(Intercept)	7.222e-05	7.120e-05	7.187e-05	1.005	0.3150				
mossSM	-1.689e-05	1.391e-05	1.430e-05	1.181	0.2376				
mossSP	1.985e-05	9.531e-06	9.979e-06	1.990	**0.0466**				
mossSS	-3.393e-05	1.504e-05	1.553e-05	2.184	**0.0290**				
TF	-7.508e-06	6.920e-06	7.026e-06	1.069	0.2853				
TP	-5.172e-07	9.467e-07	9.909e-07	0.522	0.6017				
TPA	-7.527e-07	9.089e-07	9.557e-07	0.788	0.4310				
MMP1 (full average coefficients)									
(Intercept)	4.877e-01	6.194e-02	6.539e-02	7.458	**<2e-16**	TP	0.8448	1	0.358
TPA	1.533e-03	4.536e-03	4.704e-03	0.326	0.744	TPA	1.6817	1	0.195
TF	6.882e-04	4.038e-03	4.272e-03	0.161	0.872	TF	0.3186	1	0.572
TP	-9.667e-05	2.421e-03	2.535e-03	0.038	0.970	moss	1.8034	3	0.614
mossSM	-3.291e-04	9.712e-03	1.015e-02	0.032	0.974				
mossSP	-3.285e-04	7.522e-03	7.876e-03	0.042	0.967				
mossSS	-2.293e-04	6.529e-03	6.912e-03	0.033	0.974				
(conditional average coefficients)									
(Intercept)	0.4877300	0.0619354	0.0653927	7.458	**<2e-16**				
TPA	0.0061255	0.0073523	0.0077623	0.789	0.430				
TF	0.0035326	0.0085806	0.0091451	0.386	0.699				
TP	-0.0004894	0.0054293	0.0056862	0.086	0.931				
mossSM	-0.0366302	0.0957600	0.1006899	0.364	0.716				
mossSP	-0.0365627	0.0705282	0.0746969	0.489	0.625				
mossSS	-0.0255234	0.0640276	0.0683612	0.373	0.709				
MMP8 (full average coefficients)									
(Intercept)	1.200e-05	6.403e-06	6.614e-06	1.814	0.0696	TP	2.5876	1	0.108
TF	-4.435e-07	5.089e-07	5.246e-07	0.846	0.3978	TPA	0.00	1	0.991
TP	-1.426e-07	2.448e-07	2.514e-07	0.567	0.5705	TF	0.2392	1	0.625
TPA	-1.065e-07	2.489e-07	2.564e-07	0.415	0.6778	moss	3.7207	3	0.293
mossSM	1.479e-07	1.831e-06	1.890e-06	0.078	0.9376				
mossSP	7.642e-09	1.180e-06	1.230e-06	0.006	0.9950				
mossSS	3.706e-07	1.411e-06	1.445e-06	0.256	0.7976				

(conditional average coefficients)									
(Intercept)	1.200e-05	6.403e-06	6.614e-06	1.814	0.0696				
TF	-7.533e-07	4.544e-07	4.838e-07	1.557	0.1194				
TP	-3.328e-07	2.766e-07	2.902e-07	1.147	0.2514				
TPA	-3.524e-07	3.439e-07	3.618e-07	0.974	0.3301				
mossSM	1.421e-06	5.512e-06	5.701e-06	0.249	0.8032				
mossSP	7.338e-08	3.656e-06	3.810e-06	0.019	0.9846				
mossSS	3.559e-06	2.785e-06	2.951e-06	1.206	0.2278				
MMP13 (full average coefficients)									
(Intercept)	1.259e-04	9.276e-05	9.520e-05	1.322	0.186	TP	1.013	1	0.314
mossSM	3.293e-06	1.889e-05	1.996e-05	0.165	0.869	TPA	0.043	1	0.836
mossSP	-4.398e-06	1.509e-05	1.596e-05	0.276	0.783	TF	0.775	1	0.379
mossSS	2.051e-05	3.015e-05	3.061e-05	0.670	0.503	moss	7.4231	3	0.06
TF	-4.498e-06	7.603e-06	7.805e-06	0.576	0.564				
TPA	-4.789e-07	1.842e-06	1.925e-06	0.249	0.803				
TP	-4.661e-07	1.759e-06	1.848e-06	0.252	0.801				
(conditional average coefficients)									
(Intercept)	1.259e-04	9.276e-05	9.520e-05	1.322	0.1861				
mossSM	8.794e-06	3.007e-05	3.187e-05	0.276	0.7826				
mossSP	-1.174e-05	2.284e-05	2.437e-05	0.482	0.6299				
mossSS	5.475e-05	2.349e-05	2.502e-05	2.188	**0.0287**				
TF	-9.294e-06	8.653e-06	9.016e-06	1.031	0.3026				
TPA	-2.325e-06	3.489e-06	3.700e-06	0.628	0.5298				
TP	-2.249e-06	3.305e-06	3.530e-06	0.637	0.5241				
TYR (full average coefficients)									
(Intercept)	1.669e-04	1.140e-04	1.167e-04	1.430	0.153	TP	0.1538	1	0.695
TF	-8.447e-06	9.257e-06	9.485e-06	0.891	0.373	TPA	0.0857	1	0.77
TPA	-1.215e-06	3.029e-06	3.141e-06	0.387	0.699	TF	0.5431	1	0.461
TP	-5.936e-07	2.020e-06	2.121e-06	0.280	0.780	moss	3.5654	3	0.312
mossSM	-2.481e-07	1.640e-05	1.757e-05	0.014	0.989				
mossSP	-5.556e-06	1.837e-05	1.911e-05	0.291	0.771				
mossSS	1.041e-05	2.714e-05	2.767e-05	0.376	0.707				
(conditional average coefficients)									
(Intercept)	1.669e-04	1.140e-04	1.167e-04	1.430	0.153				
TF	-1.293e-05	8.556e-06	8.931e-06	1.448	0.148				
TPA	-4.423e-06	4.385e-06	4.662e-06	0.949	0.343				
TP	-2.372e-06	3.476e-06	3.709e-06	0.639	0.523				
mossSM	-1.428e-06	3.932e-05	4.214e-05	0.034	0.973				
mossSP	-3.198e-05	3.313e-05	3.545e-05	0.902	0.367				
mossSS	5.991e-05	3.569e-05	3.799e-05	1.577	0.115				
HAS1 (full average coefficients)									
(Intercept)	1.909e-05	7.271e-06	7.534e-06	2.534	0.0113	TP	0.0637	1	0.801

TPA	-3.286e-07	5.008e-07	5.147e-07	0.638	0.5232	TPA	4.076	1	0.044
TP	-2.582e-08	2.260e-07	2.354e-07	0.110	0.9126	TF	3.0066	1	0.083
TF	-9.740e-08	5.438e-07	5.612e-07	0.174	0.8622	moss	4.8787	3	0.181
mossSM	2.736e-09	1.146e-06	1.192e-06	0.002	0.9982				
mossSP	-1.125e-07	1.141e-06	1.172e-06	0.096	0.9235				
mossSS	4.841e-08	1.124e-06	1.161e-06	0.042	0.9668				
(conditional average coefficients)									
(Intercept)	1.909e-05	7.271e-06	7.534e-06	2.534	**0.0113**				
TPA	-7.465e-07	5.077e-07	5.383e-07	1.387	0.1656				
TP	-1.033e-07	4.430e-07	4.622e-07	0.223	0.8232				
TF	-4.103e-07	1.057e-06	1.095e-06	0.375	0.7078				
mossSM	1.387e-07	8.160e-06	8.486e-06	0.016	0.9870				
mossSP	-5.702e-06	5.847e-06	6.143e-06	0.928	0.3533				
mossSS	2.454e-06	7.623e-06	7.905e-06	0.311	0.7562				
HAS2 (full average coefficients)									
(Intercept)	2.559e-02	5.973e-03	6.327e-03	4.044	**5.25e-05**	TP	0.6224	1	0.43
mossSM	-4.857e-03	3.523e-03	3.592e-03	1.352	0.176	TPA	0.4655	1	0.495
mossSP	-4.539e-03	3.120e-03	3.170e-03	1.432	0.152	TF	0.1416	1	0.707
mossSS	-3.068e-03	2.376e-03	2.442e-03	1.256	0.209	moss	16.1623	3	**0.001**
TP	-4.396e-05	2.389e-04	2.453e-04	0.179	0.858				
TPA	-6.799e-05	2.042e-04	2.113e-04	0.322	0.748				
TF	-3.773e-05	4.538e-04	4.848e-04	0.078	0.938				
(conditional average coefficients)									
(Intercept)	0.0255878	0.0059726	0.0063272	4.044	5.25e-05				
mossSM	-0.0066195	0.0022914	0.0024320	2.722	**0.006492**				
mossSP	-0.0061856	0.0017539	0.0018731	3.302	**0.000959**				
mossSS	-0.0041809	0.0017429	0.0018636	2.243	**0.024868**				
TP	-0.0001934	0.0004714	0.0004858	0.398	0.690462				
TPA	-0.0003497	0.0003405	0.0003620	0.966	0.334018				
TF	-0.0002871	0.0012229	0.0013101	0.219	0.826553				
HAS3 (full average coefficients)									
(Intercept)	6.605e-04	1.357e-04	1.415e-04	4.669	**0.000003**	TP	0.0063	1	0.937
TPA	-1.026e-05	1.038e-05	1.062e-05	0.966	0.334	TPA	2.7902	1	0.095
TP	-1.984e-06	4.884e-06	5.033e-06	0.394	0.693	TF	0.0313	1	0.86
TF	-3.357e-06	9.956e-06	1.036e-05	0.324	0.746	moss	5.2756	3	0.153
mossSM	-2.945e-06	3.134e-05	3.268e-05	0.090	0.928				
mossSP	-6.831e-06	3.138e-05	3.224e-05	0.212	0.832				
mossSS	-4.483e-06	2.668e-05	2.772e-05	0.162	0.872				
(conditional average coefficients)									
(Intercept)	6.605e-04	1.357e-04	1.415e-04	4.669	**0.000003**				
TPA	-1.699e-05	8.002e-06	8.515e-06	1.995	**0.046**				
TP	-6.330e-06	6.970e-06	7.301e-06	0.867	0.386				

TF	-1.240e-05	1.594e-05	1.687e-05	0.735	0.462
mossSM	-3.570e-05	1.036e-04	1.085e-04	0.329	0.742
mossSP	-8.282e-05	7.511e-05	7.943e-05	1.043	0.297
mossSS	-5.435e-05	7.692e-05	8.128e-05	0.669	0.504

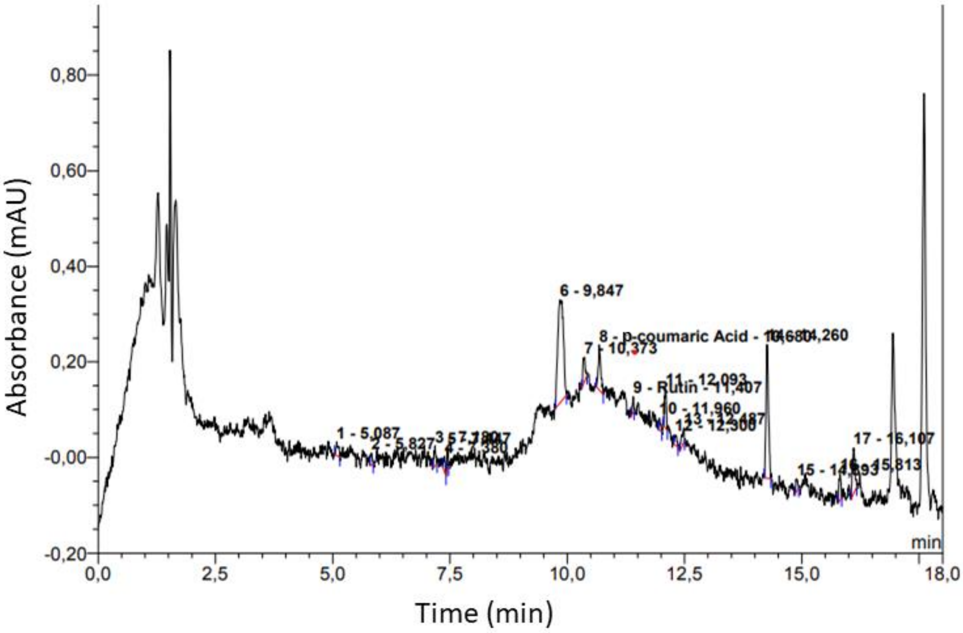

Figure S1. Chromatogram of *Sphagnum girgensohnii* Russow. (0.141 mg/g *p*-coumaric acid, 0.064 mg/g rutin).

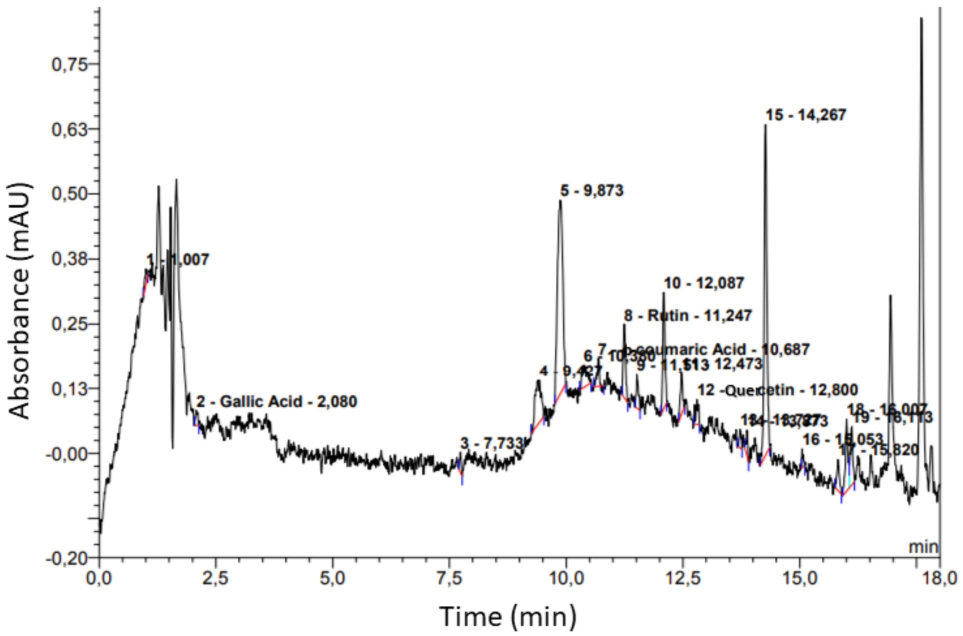

Figure S2. Chromatogram of *Sphagnum magellanicum* Brid. (0.069 mg/g *p*-coumaric acid, 0.510 mg/g rutin, 0.142 mg/g quercetin).

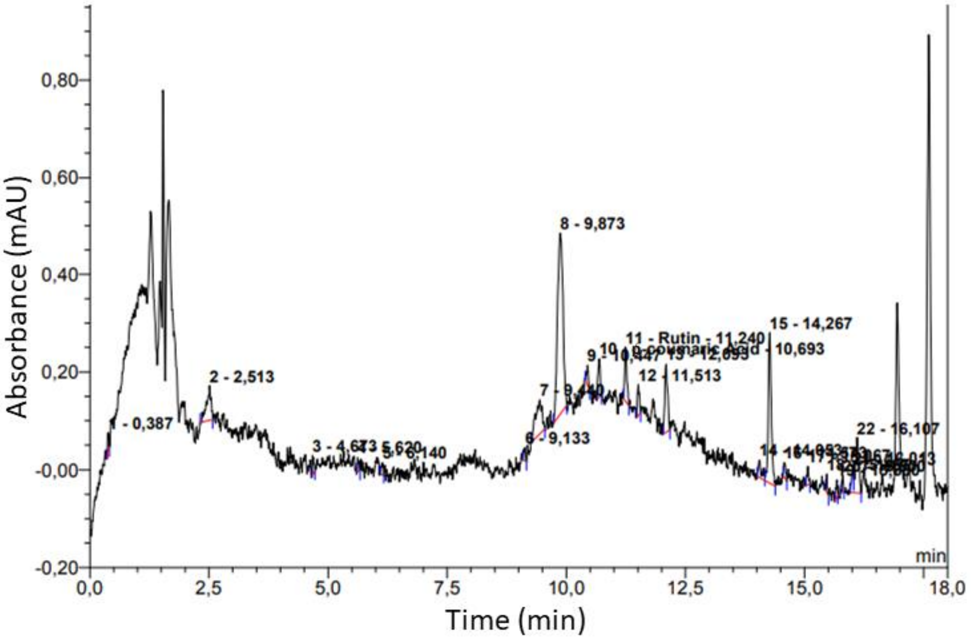

Figure S3. **Chromatogram of *Sphagnum palustre* L. (0.104 mg/g *p*-coumaric acid, 0.364 mg/g rutin).**

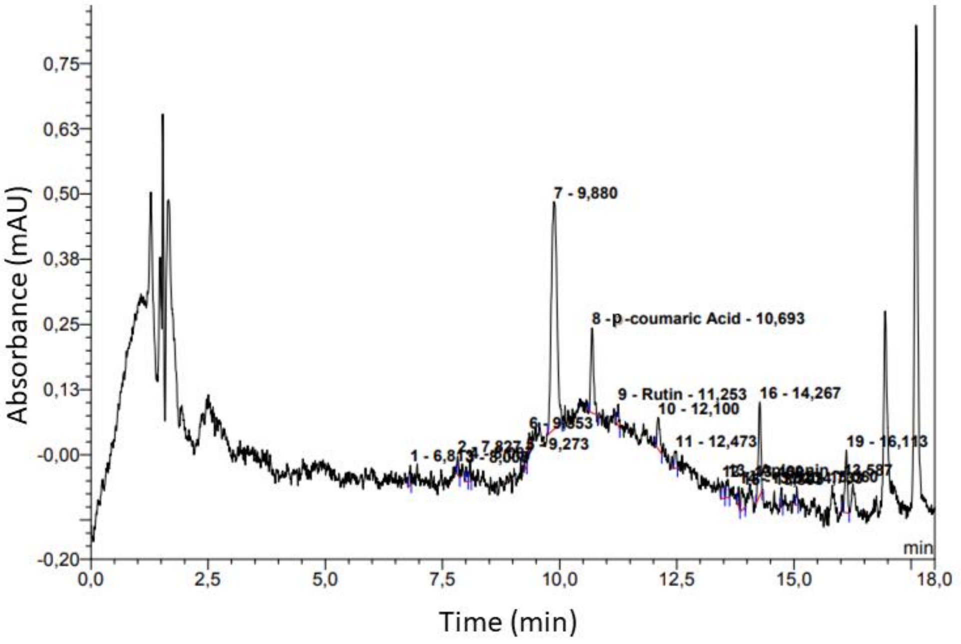

Figure S4. **Chromatogram of *Sphagnum squarrosum* Crome (0.263 mg/g *p*-coumaric acid, 0.102 mg/g rutin, 0.053 mg/g apigenin).**
